# The effects of bed rest on cardiometabolic health: A systematic review and meta‐analysis

**DOI:** 10.1113/EP092944

**Published:** 2026-03-19

**Authors:** Konstantinos Prokopidis, Tyler Daubrah‐Scott, Alyssa Varanoske, Jordi Morwani‐Mangnani, David D. Church, Colleen S. Deane, Bethan E. Phillips, Emily J. Arentson‐Lantz

**Affiliations:** ^1^ Department of Musculoskeletal Biology Institute of Life Course and Medical Sciences University of Liverpool Liverpool UK; ^2^ Centre of Metabolism, Ageing & Physiology MRC Versus Arthritis Centre for Musculoskeletal Ageing Research Nottingham NIHR Biomedical Research Centre University of Nottingham Nottingham UK; ^3^ KBR Inc. Houston Texas USA; ^4^ Leiden University Medical Centre Leiden The Netherlands; ^5^ Department of Geriatrics Donald W. Reynolds Institute on Aging Center for Translational Research in Aging and Longevity University of Arkansas for Medical Sciences Little Rock Arkansas USA; ^6^ Human Development & Health Faculty of Medicine University of Southampton Southampton General Hospital Southampton UK; ^7^ Department of Nutrition Sciences and Health Behavior University of Texas Medical Branch Galveston Texas USA

**Keywords:** bed rest, cardiometabolic health, insulin resistance, lipid profile, muscle disuse

## Abstract

Horizontal bed rest (HBR) and head‐down tilt (HDT) are models of physical inactivity. In this systematic review and meta‐analysis, we aimed to quantify changes in cardiometabolic outcomes during HBR and HDT in healthy adults. Following the PRISMA guidelines, we searched PubMed, Scopus, Web of Science and Cochrane Library until 1 October 2025. Included studies recruited healthy adults (≥18 years of age) undergoing ≥2 days of HBR or HDT. A meta‐analysis with mean differences (MD) and 95% confidence intervals (CI), using random‐effects modelling was conducted. Forty‐four studies were included. HBR (≤14 days) reduced fasting glucose (MD, −0.15 mmol/L, *P* = 0.00033). HBR (≤14 days) and HDT (≤60 days) increased insulin (HBR: MD, 7.07 pmol/L; HDT: MD, 9.65 pmol/L; *P *< 0.0001). The homeostatic model assessment for insulin resistance was increased under both models (HBR ≤30 days: MD, 0.10, *P* = 0.0004; HDT ≤60 days: MD, 0.24, *P *< 0.0001). HDT lowered total cholesterol (MD: −13.89 mg/dL, *P* = 0.000467); HBR raised triglycerides (MD, 18.96 mg/dL, *P *< 0.0001), and high‐density lipoprotein cholesterol was decreased in both models (HBR ≤14 days: MD, −4.53 mg/dL, *P* = 0.000608; HDT ≤60 days: MD, −6.89; *P *< 0.0001). C‐Reactive protein was elevated in HBR (MD, 1.57; *P *< 0.0001), and tumour necrosis factor alpha was elevated in both (HBR ≤14 days: MD, 1.33, *P *< 0.0001; HDT ≤21 days: MD, 0.40; *P* = 0.00643). In conclusion, HBR and HDT induce model‐ and duration‐specific changes in glucose metabolism and lipid profiles. Interventions that could mitigate these effects may be warranted.

## Abstract (highlights)

- **What is the central question of this study?**
  This systematic review and meta‐analysis examined cardiometabolic changes in healthy adults during horizontal bed rest (HBR) and head‐down tilt (HDT) of different durations.
- **What is the main finding and its importance?**
  Analysing 44 studies, HBR reduced fasting glucose and increased fasting insulin, homeostatic model assessment for insulin resistance and triglycerides. HDT lowered total cholesterol but increased insulin and homeostatic model assessment for insulin resistance, and both reduced high‐density lipoprotein cholesterol. C‐Reactive protein increased with HBR, and tumour necrosis factor alpha increased with both models. These findings highlight metabolic disruption from due to muscle disuse. Future relevant studies should involve more women and account for dietary intake.

## 1 INTRODUCTION

Horizontal bed rest (HBR) is a well‐established model for elucidating the negative physiological consequences of extended periods of skeletal muscle disuse (Kehler et al., 2019), as seen in situations of hospitalization or illness. Likewise, 6° head‐down tilt (HDT) bed rest is used as a ground‐based analogue of the physiological changes experienced during spaceflight (Watenpaugh, 2016). Both HBR and HDT protocols require participants to undergo several days to weeks of strict bed rest, during which they are not allowed to ambulate. Using heathy participants from across the lifespan in bed rest studies is crucial for disentangling the negative impact of disuse/unloading from the effects of (e.g.) disease or illness in hospitalized adults, or the environmental effects of spaceflight (i.e., radiation). One of the best‐described outcomes of extended periods of disuse is the rapid atrophy of skeletal muscle and subsequent loss of strength, particularly in lower limbs; this has been reviewed extensively in prior publications (Di Girolamo et al., 2021; Hardy et al., 2022; Marusic et al., 2021). In brief, measurable declines in lean soft tissue during bed rest or unloading studies are observed within 2–7 days (Arentson‐Lantz et al., 2020; Dirks et al., 2016; Drummond et al., 2013; Kilroe et al., 2019; Tanner et al., 2015) and are thought to be driven, in part, by decreases in muscle protein synthesis (Biolo et al., 2004; Brook et al., 2022; Kilroe et al., 2020; Shur et al., 2024), although it is worth noting that disuse‐induced losses in muscle mass and muscle protein synthesis are variable. More specifically, characteristics such as age, sex and duration of the disuse period have each been shown to influence the magnitude of muscle mass loss (Coffey et al., 2023; Di Girolamo et al., 2021). Given these differences, age is an important moderator.

In addition to the well‐reported detrimental effect on skeletal muscle, extended and even acute periods of disuse or unloading may also induce pronounced maladaptation to cardiometabolic health. For instance, a single day of sitting led to a 39% reduction in whole‐body insulin response in healthy adults (Stephens et al., 2011), and an increased duration (5 days) can result in as high as a 67% increase in insulin responses to glucose loading (Hamburg et al., 2007). Although there is yet to be a standard consensus on cardiometabolic health, in the context of the present manuscript, cardiometabolic health refers to factors pertinent to blood pressure, glycaemic, lipoprotein and inflammatory profiles, which are associated with chronic disease risk, including type 2 diabetes and atherosclerosis, which can result in cardiovascular disease. Alterations to the cardiovascular system during disuse include reduced microvascular and macrovascular function, decreased plasma volume, reduced stroke volume and increased resting heart rate, which may contribute to orthostatic intolerance and decreased aerobic capacity upon resumption of ambulation (Bleeker et al., 2005; Hedge et al., 2023; Rytter et al., 2020). Metabolic derangements associated with bed rest include reduced glucose uptake owing to insulin resistance (Dirks et al., 2016), in addition to an accumulation of central adiposity (Eggelbusch et al., 2024).

Middle‐aged and older adults (≥60 years) are particularly vulnerable to rapid loss of muscle mass and the development of cardiometabolic disease(s) (Cruz‐Jentoft et al., 2019). Older adults can exhibit a more profound response to disuse, with muscle atrophy, impaired glucose regulation and alterations in lipid metabolism occurring much more profoundly in comparison to younger, more metabolically flexible adults. Older populations are also more likely to be hospitalized for prolonged durations (Arentson‐Lantz et al., 2020; English et al., 2016) and are known to have a reduced capacity to regain muscle mass following disuse (Moreira et al., 2016). Additionally, older adults are also at a greater risk of chronic disease, which can be exacerbated by disuse. Given these differences, age is often viewed as a key moderator in the understanding of physiology following disuse. The metabolic flexibility of younger populations often recovers more rapidly owing to lower risk of sarcopenia and other metabolic comorbidities. Recent studies in younger populations have highlighted that bed rest of ≤3 days negatively affects muscle metabolism and insulin‐stimulated leg glucose uptake, and despite this, muscle metabolism appears to have a rebound upon remobilization, although insulin resistance can be maintained (Shur et al., 2024).

For the purpose of this review, we broadly define cardiometabolic health to encompass metabolic (i.e., glucose, insulin and lipids), inflammatory [i.e., interluekin‐6 (IL‐6), tumour necrosis factor alpha (TNF‐a) and C‐reactive protein (CRP)] and cardiovascular (blood pressure) markers. Although more complete estimates of cardiorespiratory fitness (such as maximal O_2_ uptake or heart rate) would represent the gold standard, these were rarely reported in eligible studies. Blood pressure was therefore used, because it was the most consistently reported variable as a proxy of cardiorespiratory function.

Although it is well established that bed rest has detrimental effects on cardiometabolic health, no recent meta‐analysis has been performed to quantify its impact (Ried‐Larsen et al., 2017). Additionally, heterogeneity in models (HBR vs. HDT) and duration of disuse make it difficult to characterize duration‐dependent patterns of changes in cardiometabolic outcomes. Therefore, the aim of this systematic review and meta‐analysis was to characterize the changes in key cardiometabolic outcomes, including blood pressure, inflammatory markers, circulating blood lipids and markers of glucose/insulin sensitivity, during various durations of HBR and HDT bed rest in healthy adults.

## 2 MATERIALS AND METHODS

This systematic review and meta‐analysis followed the PRISMA guidelines (Page et al., 2021) and was registered in PROSPERO. Two independent reviewers (K.P. and T.D.‐S.) conducted a search of PubMed, Scopus, Web of Science and the Cochrane Library from inception until 1 October 2025. Furthermore, reference lists of included articles were hand‐searched, and an English language restriction was applied. A detailed search strategy and keywords used are provided in Table 1.

**TABLE 1:** Search terms used in the screening of the literature search.

Database	Search terms
PubMed	(“hypokinesia” OR “muscle disuse” OR “disuse atrophy” OR “bed rest” OR “bedridden” OR “bedrest” OR “head‐down tilt”) AND (“CRP” or “C‐reactive protein” OR “hs‐CRP” OR “hsCRP” OR “high sensitivity c‐reactive protein” OR “IL‐6” OR “interleukin‐6” OR “tumour necrosis factor‐alpha” OR “TNF‐a” OR “TNF‐α” OR “lipid profile” OR “lipoprotein*” OR “LDL” OR “HDL” OR “total cholesterol” OR “serum cholesterol” OR “triglycerides” OR “insulin resistance” OR “HOMA‐IR” OR “plasma glucose” OR “serum glucose” OR “glucose disposal” OR “glucose uptake” OR “GLUT4” OR “insulin‐regulated glucose transporter” OR “glucose transporter” OR “fasting blood glucose” OR “fasting glucose” OR “fasting insulin” OR “diastolic blood pressure” OR “systolic blood pressure” OR “blood pressure”)
Cochrane Library	(“hypokinesia” OR “muscle disuse” OR “disuse atrophy” OR “bed rest” OR “bedridden” OR “bedrest” OR “head‐down tilt”) AND (“CRP” or “C‐reactive protein” OR “hs‐CRP” OR “hsCRP” OR “high sensitivity c‐reactive protein” OR “IL‐6” OR “interleukin‐6” OR “tumour necrosis factor‐alpha” OR “TNF‐a” OR “TNF‐α” OR “lipid profile” OR “lipoprotein*” OR “LDL” OR “HDL” OR “total cholesterol” OR “serum cholesterol” OR “triglycerides” OR “insulin resistance” OR “HOMA‐IR” OR “plasma glucose” OR “serum glucose” OR “glucose disposal” OR “glucose uptake” OR “GLUT4” OR “insulin‐regulated glucose transporter” OR “glucose transporter” OR “fasting blood glucose” OR “fasting glucose” OR “fasting insulin” OR “diastolic blood pressure” OR “systolic blood pressure” OR “blood pressure”)
Web of Science	(“hypokinesia” OR “muscle disuse” OR “disuse atrophy” OR “bed rest” OR “bedridden” OR “bedrest” OR “head‐down tilt”) AND (“CRP” or “C‐reactive protein” OR “hs‐CRP” OR “hsCRP” OR “high sensitivity c‐reactive protein” OR “IL‐6” OR “interleukin‐6” OR “tumour necrosis factor‐alpha” OR “TNF‐a” OR “TNF‐α” OR “lipid profile” OR “lipoprotein*” OR “LDL” OR “HDL” OR “total cholesterol” OR “serum cholesterol” OR “triglycerides” OR “insulin resistance” OR “HOMA‐IR” OR “plasma glucose” OR “serum glucose” OR “glucose disposal” OR “glucose uptake” OR “GLUT4” OR “insulin‐regulated glucose transporter” OR “glucose transporter” OR “fasting blood glucose” OR “fasting glucose” OR “fasting insulin” OR “diastolic blood pressure” OR “systolic blood pressure” OR “blood pressure”)
Scopus	(“hypokinesia” OR “muscle disuse” OR “disuse atrophy” OR “bed rest” OR “bedridden” OR “bedrest” OR “head‐down tilt”) AND (“CRP” or “C‐reactive protein” OR “hs‐CRP” OR “hsCRP” OR “high sensitivity c‐reactive protein” OR “IL‐6” OR “interleukin‐6” OR “tumour necrosis factor‐alpha” OR “TNF‐a” OR “TNF‐α” OR “lipid profile” OR “lipoprotein*” OR “LDL” OR “HDL” OR “total cholesterol” OR “serum cholesterol” OR “triglycerides” OR “insulin resistance” OR “HOMA‐IR” OR “plasma glucose” OR “serum glucose” OR “glucose disposal” OR “glucose uptake” OR “GLUT4” OR “insulin‐regulated glucose transporter” OR “glucose transporter” OR “fasting blood glucose” OR “fasting glucose” OR “fasting insulin” OR “diastolic blood pressure” OR “systolic blood pressure” OR “blood pressure”)
	(“hypokinesia” OR “muscle disuse” OR “disuse atrophy” OR “bed rest” OR “bedridden” OR “bedrest” OR “head‐down tilt”) AND (“CRP” or “C‐reactive protein” OR “hs‐CRP” OR “hsCRP” OR “high sensitivity c‐reactive protein” OR “IL‐6” OR “interleukin‐6” OR “tumour necrosis factor‐alpha” OR “TNF‐a” OR “TNF‐α” OR “lipid profile” OR “lipoprotein*” OR “LDL” OR “HDL” OR “total cholesterol” OR “serum cholesterol” OR “triglycerides” OR “insulin resistance” OR “HOMA‐IR” OR “plasma glucose” OR “serum glucose” OR “glucose disposal” OR “glucose uptake” OR “GLUT4” OR “insulin‐regulated glucose transporter” OR “glucose transporter” OR “fasting blood glucose” OR “fasting glucose” OR “fasting insulin” OR “diastolic blood pressure” OR “systolic blood pressure” OR “blood pressure”)

### 2.1 Inclusion and exclusion criteria

Studies were included if they met the following criteria: (1) were clinical trials involving healthy or community‐dwelling adults aged ≥18 years; (2) involved whole‐body muscle disuse, such as a minimum HBR or HDT duration of 2 days; (3) reported of one or more of the following pre and post outcomes: systolic blood pressure (SBP), diastolic blood pressure (DBP), homeostatic model assessment for insulin resistance (HOMA‐IR), fasting glucose/insulin, total cholesterol (TC), triglycerides, low‐density lipoprotein cholesterol (LDL‐C), high‐density lipoprotein cholesterol (HDL‐C), CRP, IL‐6 and TNF‐a; and (4) controlled for diet.

Studies were excluded if they: (1) included participants who were not healthy or community‐dwelling, or were <18 years or undergoing surgery; (2) were reviews, letters, commentaries, or in vivo/in vitro experiments; (3) were not full‐text publications; (4) were not written in English; (5) did not provide sufficient data to extract pre‐ and post‐intervention outcomes; (6) if baseline assessments were conducted >1 day prior to the start of the intervention, to maximize temporal consistency between baseline and intervention onset across studies (although day‐to‐day variability cannot be eliminated fully, this criterion was applied to reduce heterogeneity arising from pre‐intervention intervals); and (7) lacked a dietary control. In this review, ‘dietary control’ is defined as any intervention whereby nutritional intake by each participant was standardized, such as provision of standardized meals or determined caloric intake (using validated formulas defined by each author), excluding studies where diets were self‐selected without adequate research team input.

### 2.2 Data extraction and risk of bias

Data were extracted independently by two co‐authors (K.P. and T.D.‐S.). Data regarding authors, year of publication, study design, health status, patient characteristics [age, sex, body mass index (BMI) or body weight, body fat mass and lean soft tissue], baseline physical activity values, whether dietary intake was assessed, units of measurement and assays used related to our outcomes of interest (cardiometabolic markers), and study duration were collected. Where reported, the method of assessing body composition was also extracted [i.e., dual‐energy X‐ray absorptiometry (DXA), bioelectrical impedance analysis (BIA), MRI, CT or skinfold callipers]. Two models of intervention were subject to the extraction phase, HBR (supine position, with no angle of tilt) and HDT (at 6° head‐down tilt, inducing cephalad shifts to simulate microgravity). For studies where missing/unclear numerical data were not readily available, contact was made to each corresponding author, with up to two contact attempts made per study. Responses were received for one of four studies for cardiometabolic parameters and 1 of 26 for physical activity data. Where data could not be obtained via author contact, WebPlotDigitizer was used to extract graphical format data (Drevon et al., 2017). To prevent data duplication and improve interpretability, intervention duration was categorized into distinct segments, unless otherwise stated: ≤7, ≤14, ≤21, ≤30and ≤60 days. When a study reported multiple time points, only the longest duration within the defined exclusion criteria (≤60 days) was used in each pooled analysis. This avoided pseudoreplication and allowed for a clearer interpretation of cardiometabolic adaptations to the intervention. For outcomes where HOMA‐IR was not reported directly, data were extracted from fasting insulin and fasting glucose. These were then input into the following expression: [fasting glucose (mmol/L) × fasting insulin (pmol/L)]/156.25. The values from this calculation were analysed in an identical manner to the directly reported HOMA‐IR values in the meta‐analysis to maximize the power of the available dataset.

The risk of bias for the included studies was evaluated using the Risk of Bias in Non‐randomized Studies of Interventions (ROBINS‐I) assessment tool (Sterne et al., 2016) by three investigators (K.P., T.D.‐S. and J.M.).

### 2.3 Statistical analysis

For statistical analysis, effect sizes were calculated as mean differences (MD) with 95% confidence intervals (CIs) from pre‐ to post‐HBR/HDT. When standard errors (SE) were extracted, they were converted to standard deviation (SD) using: SE × square root (of the sample size); ΔSDs were computed using baseline and final SDs with an assumed correlation coefficient of 0.7, following the Cochrane Handbook formula, when correlations were unavailable. When measurement units in the outcomes of interest could not be converted uniformly among studies, standardized MD (SMD) was used. Statistical significance was determined using random effects modelling and the inverse‐variance method. Heterogeneity across studies was assessed by the overlap of CIs and tested using Cochran's *Q* (χ^2^ test) and *I* ^2^. Data were classified as moderately heterogeneous (*I* ^2^ = 50%–74.9%) or highly heterogeneous (*I* ^2^ ≥ 75%). Subgroup analyses based on mean age and study duration were conducted where data were available. Sensitivity analyses were conducted based on studies including only men or women, and studies with higher risk of bias. Publication bias was evaluated with Begg's funnel plots and Egger's linear regression test, and meta‐regression of baseline BMI/body weight was performed for outcomes involving ≥10 studies to examine its potential as a moderator (Mathur & VanderWeele, 2021). The meta‐analysis was conducted using R software, with the ‘metafor’ package (RStudio v.2025.09.1+401).

## 3 RESULTS

The initial literature search provided 7535 publications. Following the exclusion of duplicates and non‐relevant abstracts that were marked as ineligible, 89 full texts were assessed for eligibility to be included in this study. Of these 89 reports, 45 were excluded using the exclusion criteria, as follows: 17 studies did not report a dietary control, seven used an identical population (or part of it) that was eventually included in this review, 11 had insufficient information around outcomes of interest, 1 included a nutritional intervention, 1 included non‐healthy adults, 1 had measured baseline values 4 days prior to bed rest, 2 induced hypercortisolaemia, 1 used a pharmaceutical intervention, 1 involved bone‐fracture patients, 1 used values only from RNA data, 1 used one‐leg immobilization, and in 1 study participants were tube fed. All excluded studies are outlined in Table 2.

**TABLE 2:** Excluded studies.

Reason for exclusion	References
No dietary control (*n* = 17)	Aletti et al. (2012); Arinell et al. (20110; Biensø et al. (20120; Burns et al. (2021); Chouker et al. (2001); Custaud et al. (2002); Drummond et al. (2013); Feuerecker et al. (2013); Hamburg et al. (2007); Hoffmann et al. (2022); Hoffmann et al. (2021); Iwasaki et al. (2005); Kanikowska et al. (2008); McGavock et al. (2009); Ryan et al. (2016); Shiraishi et al. (2003); Trudel et al. (2009)
Insufficient information (*n* = 11)	Blaber et al. (2023); Coupé et al. (2011); Eiken et al. (2008); Fortrat et al. (2001); Hesse et al. (2005); Hoenemann et al. (2024); Kiilerich et al. (2011); Mutin‐Carnino et al. (2014); Pavy‐Le Traon et al. (1997); Reidy et al. (2020); Taylor et al. (1949)
Identical populations of included studies (*n* = 7)	Hirayanagi et al. (2004); Hodges et al. (2010); Mahmassani et al. (2019); Möstl et al. (2021); Sonne et al. (2010); Traon et al. (2002); Yang et al. (2011)
Hypercortisolaemia was induced (*n* = 2)	Cree et al. (2005); Mangogna et al. (2023)
Non‐healthy (*n* = 1)	Koo et al. (2017)
Nutritional intervention (*n* = 1)	Kelsen et al. (2012)
Measured baseline values 4 days prior to bed rest (*n* = 1)	Beck et al. (1992)
Pharmacological intervention (*n* = 1)	Arzeno et al. (2013)
Bone‐fractured patients (*n* = 1)	Myllynen et al. (1987)
One‐leg immobilization (*n* = 1)	Wall et al. (2015)
Measured glucose excretion (*n* = 1)	Trim et al. (2023)
Nursing home tube fed (*n* = 1)	Hsieh et al. (2010)

**FIGURE 1:**
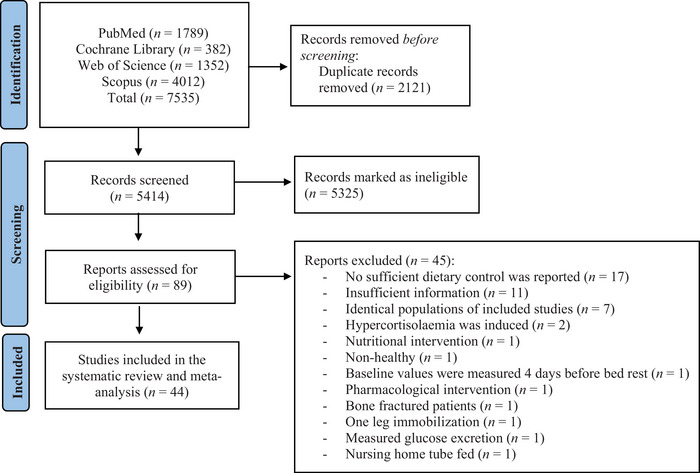
PRISMA flowchart.

In total, 44 studies were included in this systematic review and meta‐analysis (Alibegovic et al., 2010; Amirova et al., 2020; Barbic et al., 2019; Blaber et al., 2023; Blanc et al., 2000; Coker et al., 2014; Convertino et al., 1990; Deutz et al., 2013; Dirks et al., 2016; Downs et al., 2020; Duran‐Valdez et al., 2008; Eggelbusch et al., 2024; Fuchs et al., 2024; Hajj‐Boutros et al., 2023; Harder‐Lauridsen et al., 2017; Heer et al., 2014; Højbjerre et al., 2010; Hönemann et al., 2024; Jurdana et al., 2015; Krachtis et al., 2022; Li et al., 2017; Liphardt et al., 2018; Mastrandrea et al., 2025; Mikines et al., 1989; Montero et al., 2018; Mulder et al., 2014; Nosova et al., 2014; Petrocelli et al., 2020; Rudwill et al., 2015, 2018; Sadeghian et al., 2022; Shangraw et al., 1988; Shi et al., 2014; Smeuninx et al., 2021; Sonne et al., 2011; Stenger et al., 2012; Trakaki et al., 2020; Ward et al., 2020, 2024; Yanagibori et al., 1998; Yang et al., 2014; Yao et al., 2012; Zorbas et al., 1992; Zwart et al., 2009). A PRISMA flowchart is shown in Figure 1. Minimum mean age was 20.4 years, and maximum mean age was 71.5 years. Minimum and maximum mean BMI were 20.9 and 42.0 kg/m^2^, respectively. Women represented 22.2% of the sample size. Eighteen studies examined HBR as an intervention, and 25 studies examined HDT. Body composition measures were reported in 26 of the 44 included studies, with the most prominent of these being DXA, whereas 18 studies did not report body composition data. Physical activity or habitual activity level prior to bed rest was reported in 9 of the 44 studies. Of these, five recorded activity/reported data via a wearable device, in a further study subjects wore a physical activity monitor but data were not reported, and three studies asked participants to refrain from strenuous exercise, alcohol and rest for 24–72 h prior to the intervention but did not record physical activity levels. Full characteristics of the included studies are presented in Table 3.

**TABLE 3:** Study and participant characteristics of the included studies.

				Participant characteristics			
Study, year	Disuse model	Intervention duration	Outcomes	*n* (male/female)	Age (years)	BMI/body weight (kg/m^2^)	Body composition tool
Alibegovic et al., 2010	HBR	9 days	SBP, DBP, glucose, insulin, HOMA‐IR, TC, triglycerides, LDL, HDL, body weight	23 (23/0)	56 ± 4.3	24.3 ± 2.3	DXA
Amirova et al. (2020)	HDT	21 days	SBP, DBP	11 (11/0)	34 ± 2	22.4 ± 0.5	–
Barbic et al., 2019	HDT	21 days	SBP, DBP	10 (10/0)	33 ± 3.2	23.4 ± 0.6	–
Blaber et al., 2023 (males)	HDT	14 days	SBP, DBP, TNF‐a, IL‐6	4 (4/0)	60 ± 5	–	–
Blaber et al., 2023 (females)	HDT	14 days	SBP, DBP, TNF‐a, IL‐6	4 (0/4)	60 ± 5	–	–
Blanc et al., 2000 (males)	HDT	7 days	Glucose, insulin	8 (8/0)	32.4 ± 2.5	23.9 ± 1.98	–
Blanc et al., 2000 (females)	HDT	7 days	Glucose, insulin	8 (0/8)	27.9 ± 2.5	20.9 ± 1.7	–
Coker et al., 2014	HBR	10 days	Glucose, body weight	8 (7/1)	64 ± 8.5	28.1 ± 4.8	DXA
Convertino et al., 1990	HDT	25 days	SBP, DBP	11 (11/0)	40 ± 3	79 ± 6.6 kg	–
Deutz et al., 2013	HBR	10 days	Glucose, CRP, TC, body weight	8 (7/1)	67.1 ± 1.7	26.5 ± 1.2	DXA
Dirks et al., 2016	HBR	7 days	Glucose	10 (10/0)	23 ± 1	23 ± 2.8	CT
Downs et al., 2020	HDT	70 days	Glucose, insulin, HOMA‐IR	9 (9/0)	38.1 ± 7.8	25.7 ± 3.6	DXA
Duran‐Valdez et al., 2008	HBR	2 days	Glucose, IL‐6, CRP	5 (1/4)	33.8 ± 6.3	42 ± 19.2	–
Eggelbusch et al., 2024	HDT	60 days	GLUT‐4, insulin, glucose, CRP, IL‐6, TNF‐a	24 (16/8)	33 ± 9	22.1 ± 6.7	DXA
Fuchs et al., 2024	HBR	14 days	Glucose, insulin, body weight	12 (12/0)	24 ± 3	23.7 ± 3.1	DXA
Hajj‐Boutros et al., 2023	HDT	14 days	TC, triglycerides, HDL, LDL, glucose, CRP, body weight	11 (11/0)	58.4 ± 3.9	24 ± 2.8	DXA
Harder‐Lauridsen et al., 2017	HBR	8 days	Glucose, insulin, SBP, DBP, TNF‐a, IL‐6, HDL, LDL, triglycerides, TC, HOMA‐IR	10 (10/0)	23 ± 3.6	23.6 ± 2.2	MRI
Heer et al., 2014	HDT	21 days	Glucose, HOMA‐IR, triglycerides, TC, CRP	7 (7/0)	27.6 ± 3.3	24.1 ± 1.9	DXA
Højbjerre et al., 2010	HBR	10 days	Glucose, insulin, GLUT‐4	20 (20/0)	25 ± 1.2	24.2 ± 2.9	DXA
Hoenemann et al., 2024	HDT	30 days	SBP, DBP	12 (7/5)	35 ± 8	23.1 ± 2.3	–
Jurdana et al., 2015 (young subjects)	HBR	14 days	TNF‐a, IL‐6, CRP, body weight	7 (7/0)	24 ± 6	24 ± 2.4	BIA
Jurdana et al., 2015 (older subjects)	HBR	14 days	TNF‐a, IL‐6, CRP, body weight	16 (16/0)	60 ± 5	26.6 ± 4.4	BIA
Krachtis et al., 2022	HDT	60 days	SBP, DBP	11 (11/0)	28.3 ± 6.3	75.2 ± 7.0 kg	–
Li et al., 2017	HDT	4 days	SBP, DBP, body weight	6 (6/0)	23 ± 3	55–67 kg	–
Liphardt et al., 2018	HDT	21 days	TNF‐a	9 (9/0)	31 ± 6.2	–	–
Mastrandrea et al., 2025	HBR	14 days	Glucose, insulin, HOMA‐IR	11 (6/5)	59 ± 3	24.9 ± 3.0	–
Mikines et al., 1989	HBR	7 days	Glucose	6 (6/0)	25 ± 1	72.2 ± 3.6 kg	Skinfold callipers
Montero et al., 2018	HBR	4 days	Glucose, body weight	13 (13/0)	23 ± 2	23.6 ± 2.4	DXA
Mulder et al., 2014	HDT	5 days	SBP, DBP	10 (10/0)	29.7 ± 1.9	63.9 ± 1.2 kg	DXA
Nosova et al., 2014	HBR	5 days	SBP, DBP, CRP, IL‐6, TNF‐a	5 (4/1)	22 ± 2	24 ± 3	DXA
Petrocelli et al., 2020 (young subjects)	HBR	5 days	HDL, LDL, TC, triglycerides, HOMA‐IR, body weight	13 (7/6)	23.4 ± 3.2	22.1 ± 3.4	DXA
Petrocelli et al., 2020 (older subjects)	HBR	5 days	HDL, LDL, TC, triglycerides, HOMA‐IR, body weight	22 (11/9)	67.8 ± 5.5	24.9 ± 2.7	DXA
Rudwill et al., 2015	HBR	30 days	HOMA‐IR, triglycerides, body weight	8 (0/8)	34 ± 1.4	21 ± 0.5	–
Rudwill et al., 2018	HDT	21 days	Glucose, insulin, CRP, GLUT‐4, LDL, HDL	9 (0/8)	31 ± 6.3	23.8 ± 1.5	DXA
Sadeghian et al., 2022	HDT	14 days	SBP, DBP	5 (5/0)	58.7 ± 0.5	70.2 ± 3.2 kg	–
Shangraw et al., 1988	HBR	7 days	Glucose, insulin	6 (6/0)	24 ± 3	–	MRI
Shi et al., 2014	HDT	29 days	SBP, DBP	7 (7/0)	32.5 ± 7.5	63 ± 3	–
Smeuninx et al., 2021	HBR	5 days	Glucose, insulin, HOMA‐IR, TC, HDL, triglycerides, body weight	10 (10/0)	71.5 ± 4	25.5 ± 2.8	BIA
Sonne et al., 2011	HDT	10 days	Glucose, HbA1c, SBP, DBP, CRP	20 (20/0)	25 ± 0.9	24.1 ± 10	DXA
Stenger et al., 2012	HDT	21 days	SBP, body weight	7 (7/0)	32 ± 6	67–95 kg	–
Trakaki et al., 2020	HDT	21 days	TC, HDL, LDL, triglycerides	11 (11/0)	34.3 ± 8.3	22.4 ± 1.7	–
Ward et al., 2020	HDT	60 days	Triglycerides, HDL, body weight	11 (11/0)	28 ± 6	23.3 ± 2	–
Ward et al., 2024	HDT	60 days	Glucose, insulin, triglycerides, TC, HDL, LDL, body weight	11 (11/0)	28 ± 6	23.3 ± 2	DXA
Yanagibori et al., 1998	HBR	20 days	HDL, LDL, TC, triglycerides, body weight	10 (5/5)	20.4 ± 1.8	Males: 69.2 ± 13.7 kg Females: 55.0 ± 4.5 kg	Skinfold callipers
Yang et al., 2014	HDT	60 days	HDL, LDL, glucose, insulin	14 (14/0)	32.5 ± 7.5	22.5 ± 2.5	–
Yao et al., 2012	HDT	4 days	SBP, DBP, body weight	6 (6/0)	21.5 ± 0.5	23 ± 3	–
Zorbas et al., 1992	HBR	30 days	Glucose, body weight	6 (6/0)	22.7 ± 4.3	70.6 ± 9.3 kg	–
Zwart et al., 2009	HDT	21 days	CRP, glucose, TC, triglycerides, body weight	7 (7/0)	28 ± 2	82.0 ± 8.0 kg	DXA

*Note*: Data are expressed as the mean (SD). Abbreviations: BIA, bioelectrical impedance; BMI, body mass index; CRP, C‐reactive protein; DBP, diastolic blood pressure; DXA, dual X‐ray absorptiometry; GLUT‐4, glucose transporter type 4; HbA1c, glycosylated haemoglobin; HBR, horizontal bed rest; HDL, high‐density lipoprotein; HDT, head‐down tilt; HOMA‐IR, homeostatic model assessment for insulin resistance; IL‐6, interleukin‐6; LDL, low‐density lipoprotein; SBP, systolic blood pressure; TC, total cholesterol; TNF‐a, tumour necrosis factor alpha.

All results from the meta‐analyses are summarized in Table 4; forest plots are shown in Supporting Information File S1 and bubble plots in Supporting Information File S2.

**TABLE 4:** Results of the meta‐analyses.

Variable	*n*	MD	95% CI	*I* ^2^ (%)	*P*‐value
Glucose, mmol/L					
Bed rest (≤7 days)	8	−0.04	−0.13 to 0.04	17	0.31
Bed rest (≤14 days)	15	−0.15	−0.20 to −0.10	61	<0.01^*^
Bed rest (≤14 days), including data with body weight changes	6	−0.29	−0.38 to −0.20	61	<0.01^*^
Head‐down tilt (≤21 days)	5	−0.01	−0.07 to 0.06	81	0.79
Head‐down tilt (≤60 days)	7	0.01	−0.05 to 0.07	74	0.77
Body weight, kg, bed rest (≤14 days)	6	−1.30	−3.05 to 0.44	0	0.14
Insulin, pmol/L					
Bed rest (≤14 days)	7	7.07	3.21 to 10.93	37	<0.01^*^
Head‐down tilt (≤21 days)	3	12.55	2.84 to 22.26	0	0.01^*^
Head‐down tilt (≤60 days)	5	9.65	3.53 to 15.77	0	<0.01^*^
HOMA‐IR					
Bed rest (≤14 days)	8	0.09	0.03 to 0.15	62	<0.01^*^
Bed rest (≤30 days)	9	0.10	0.05 to 0.16	67	<0.01^*^
Bed rest (≤30 days), including data with BMI changes	4	0.26	0.16 to 0.36	0	<0.01^*^
Head‐down tilt (≤21 days)	4	0.09	−0.05 to 0.23	21	0.21
Head‐down tilt (≤60 days)	7	0.24	0.15 to 0.33	50	<0.01^*^
BMI, kg/m^2^, bed rest (≤30 days)	4	−0.74	−1.28 to −0.20	0	<0.01^*^
Total cholesterol, mg/dL					
Bed rest (≤10 days)	6	−2.09	−6.79 to 2.62	48	0.39
Head‐down tilt (≤21 days)	3	−18.63	−27.25 to −10.01	0	<0.01^*^
Head‐down tilt (≤60 days)	4	−13.89	−21.67 to −6.11	55	<0.01^*^
Triglycerides, mg/dL					
Bed rest (≤10 days)	5	15.12	5.24 to 24.99	14	<0.01^*^
Bed rest (≤20 days)	6	18.45	9.44 to 27.47	31	<0.01^*^
Bed rest (≤30 days)	7	18.96	10.26 to 27.67	20	<0.01^*^
Bed rest (≤30 days), including data with BMI changes	4	14.91	3.36 to 26.46	0	0.01^*^
Head‐down tilt (≤21 days)	3	−14.29	−22.57 to −6.01	73	<0.01^*^
Head‐down tilt (≤60 days)	4	−6.85	−13.84 to 0.13	84	0.05
BMI, kg/m^2^, bed rest (≤30 days)	4	−0.74	−1.28 to −0.20	0	<0.01^*^
High‐density lipoprotein, mg/dL					
Bed rest (≤14 days)	6	−4.53	−7.12 to −1.94	33	<0.01^*^
Head‐down tilt (≤60 days)	3	−6.89	−9.41 to −4.36	0	<0.01^*^
Low‐density lipoprotein, mg/dL					
Bed rest (≤14 days)	5	0.83	−1.59 to 3.26	31	0.50
Head‐down tilt (≤60 days)	3	−6.15	−13.31 to 1.01	71	0.09
Systolic blood pressure, mmHg					
Bed rest (≤10 days)	4	−1.83	−4.27 to 0.61	0	0.14
Head‐down tilt (≤14 days)	8	0.88	−8.16 to 9.93	95	0.85
Head‐down tilt (≤30 days)	12	2.20	−3.87 to 8.28	93	0.48
Diastolic blood pressure, mmHg					
Bed rest (≤10 days)	4	0.51	−1.42 to 2.44	46	0.60
Head‐down tilt (≤14 days)	8	−0.28	−1.61 to 1.05	92	0.68
Head‐down tilt (≤30 days)	11	1.43	−2.29 to 5.16	91	0.45
C‐Reactive protein, mg/L					
Bed rest (≤14 days)	4	1.57	0.91 to 2.24	91	<0.01^*^
Head‐down tilt (≤55 days)	3	0.05	−0.04 to 0.13	58	0.27
Interleukin‐6, pg/mL					
Bed rest (≤14 days)	4	0.12	−0.09 to 0.32	96	0.28
Tumour necrosis factor alpha, pg/mL					
Bed rest (≤14 days)	3	1.33	1.04 to 1.62	94	<0.01^*^
Head‐down tilt (≤21 days)	3	0.40	0.11 to 0.68	0	<0.01^*^

Abbreviations: BMI, body mass index; CI, confidence interval; HOMA‐IR, homeostatic model assessment for insulin resistance; MD, mean difference.

^*^ *P* < 0.05 indicates significance.

### 3.1 Effects of HBR and HDT on glucose, insulin and HOMA‐IR

Up to 7 days of HBR did not significantly impact fasting glucose (*n* = 8; MD: −0.04, 95% CI: −0.13 to 0.04 mmol/L, *I* ^2 ^= 17%, *P* = 0.312). Longer‐duration HBR, ≤14 days, induced a modest but significant reduction in glucose (*n* = 15; MD: −0.15, 95% CI: −0.20 to −0.10 mmol/L, *I* ^2^ = 61%, *P* = 0.00033). The changes in fasting glucose were not associated with changes in body weight (*n* = 6; MD: −1.30, 95% CI: −3.05 to 0.44 kg, *I* ^2 ^= 0%, *P* = 0.143). No changes in fasting glucose were detected in HDT studies lasting ≤21 days (*n* = 5; MD: −0.01, 95% CI: −0.07 to 0.06 mmol/L, *I* ^2 ^= 81%, *P* = 0.792) or ≤60 days (*n* = 7; MD: 0.01, 95% CI: −0.05 to 0.07 mmol/L, *I* ^2 ^= 74%, *P* = 0.767).

Fasting insulin was significantly increased following HBR of ≤14 days (*n* = 7; MD: 7.07, 95% CI: 3.21 to 10.93 pmol/L, *I* ^2 ^= 37%, *P* < 0.0001). Likewise, ≤21 days of HDT contributed to a significant increase in fasting insulin (*n* = 3; MD: 12.55, 95% CI: 2.84 to 22.26 pmol/L, *I* ^2 ^= 0%, *P* = 0.0113), and it also increased following ≤60 days of HDT (*n* = 5; MD: 9.65, 95% CI: 3.53 to 15.77 pmol/L, *I* ^2 ^= 0%, *P* = 0.00201).

Up to 14 days of HBR significantly increased HOMA‐IR (*n* = 8; MD: 0.09, 95% CI: 0.03 to 0.15, *I* ^2 ^= 62%, *P* = 0.00277). HOMA‐IR was also significantly increased following ≤30 days of HBR (*n* = 9; MD: 0.10, 95% CI: 0.05 to 0.16, *I* ^2 ^= 67%, *P* = 0.0004). Including data with BMI changes, this increase remained significant (*n* = 4; MD: 0.26, 95% CI: 0.16 to 0.36, *I* ^2 ^= 0%, *P* < 0.0001). HOMA‐IR did not change in HDT ≤21 days (*n* = 4; MD: 0.09, 95% CI: 0.05 to 0.23, *I* ^2 ^= 21%, *P* = 0.208), but HOMA‐IR was significantly increased following HDT of ≤60 days (*n* = 7; MD: 0.24, 95% CI: 0.15 to 0.33, *I* ^2 ^= 50%, *P* < 0.0001).

### 3.2 Effects of HBR and HDT on total cholesterol, triglycerides, HDL and LDL cholesterol

TC did not change significantly in response to ≤10 days of HBR (*n* = 6, MD: −2.09, 95% CI: ‐6.79 to 2.62 mg/dL, *I* ^2 ^= 48%, *P* = 0.385). Up to 21 days of HDT significantly decreased TC (*n* = 3; MD: −18.63, 95% CI: −27.25 to −10.01 mg/dL, *I* ^2 ^= 0%, *P* < 0.0001), a significant change that was also seen in HDT studies of ≤60 days (*n* = 4; MD: −13.89, 95% CI: −21.67 to −6.11 mg/dL, *I* ^2 ^= 55%, *P* = 0.000467).

Triglycerides significantly increased following HBR of ≤10 days (*n* = 5; MD: 15.12, 95% CI: 5.24–24.99 mg/dL, *I* ^2 ^= 14%, *P* = 0.0027), ≤20 days (*n* = 6; MD: 18.45, 95% CI: 9.44–27.47 mg/dL, *I* ^2 ^= 31%, *P* < 0.0001) and ≤30 days (*n* = 7; MD: 18.96, 95% CI: 10.26–27.67 mg/dL, *I* ^2 ^= 20%, *P* < 0.0001). The changes in triglycerides seen in HBR were significantly associated with changes in BMI (*n* = 4; MD: 14.91, 95% CI: 3.36–26.46 kg, *I* ^2 ^= 0%, *P* = 0.0114). HDT of ≤21 days significantly increased triglycerides (*n* = 3; MD: −14.29, 95% CI: −22.57 to −6.01 mg/dL, *I* ^2 ^= 73%, *P* = 0.00721), but no changes in triglycerides in response to ≤60 days of HDT were observed (*n* = 4; MD: −6.85, 95% CI: −13.84 to 0.13 mg/dL, *I* ^2 ^= 84%, *P* = 0.0545).

HDL‐C significantly decreased following ≤14 days of HBR (*n* = 6, MD: −4.53, 95% CI: −7.14 to −1.94 mg/dL, *I* ^2 ^= 33%, *P* = 0.000608). A similar pattern was seen in the HDT model following durations of ≤60 days of HDT (*n* = 3; MD: −6.89, 95% CI: −9.41 to −4.36 mg/dL, *I* ^2 ^= 0%, *P* < 0.0001).

LDL‐C did not change in response to any duration of HBR or HDT.

### 3.3 Effects of HBR and HDT on systolic and diastolic blood pressure

No changes were observed following HBR of ≤10 days for either SBP (*n* = 4; MD: −1.83, 95% CI: −4.27 to 0.61 mmHg, *I* ^2 ^= 0%, *P* = 0.141) or DBP (*n* = 4; MD: 0.51, 95% CI: –1.42 to 2.44 mmHg, *I* ^2 ^= 46%, *P* = 0.603). Likewise, no changes were shown with HDT of ≤14 days (SPB: *n* = 8; MD: 0.88, 95% CI: 8.16 to 9.93 mmHg, *I* ^2 ^= 95%, *P* = 0.848; and DBP: *n* = 8; MD: −0.28, 95% CI: −1.61 to 1.05 mmHg, *I* ^2 ^= 92%, *P* = 0.682) or ≤30 days (SPB: *n* = 12; MD: 2.20, 95% CI: −3.87 to 8.28 mmHg, *I* ^2 ^= 93%, *P* = 0.477; and DBP: *n* = 11; MD: 1.43, 95% CI: −2.29 to 5.16 mmHg, *I* ^2 ^= 91%, *P* = 0.451).

### 3.4 Effects of HBR and HDT on CRP, IL‐6 and TNF‐a

Plasma CRP levels increased significantly following ≤14 days of HBR (*n* = 4; MD: 1.57, 95% CI: 0.91 to 2.24, *I* ^2^ = 91%, *P* < 0.0001), but no significant change was observed with ≤55 days of HDT (*n* = 3; MD: 0.05, 95% CI: −0.04 to 0.13, *I* ^2^ = 58%, *P* = 0.274). Likewise, no changes were observed in regards to IL‐6 following ≤14 days of HBR (*n* = 4; MD: 0.12, 95% CI: −0.09 to 0.32, *I* ^2^ = 96%, *P* = 0.277); however, TNF‐a increased significantly following ≤14 days of HBR (*n* = 3; MD: 1.33, 95% CI: 1.04 to 1.62, *I* ^2^ = 94%, *P* < 0.0001) and ≤21 days of HDT (*n* = 3; MD: 0.40, 95% CI: 0.11 to 0.68, *I* ^2^ = 0%, *P* = 0.00643).

### 3.5 Publication bias and meta‐regressions

**FIGURE 2:**
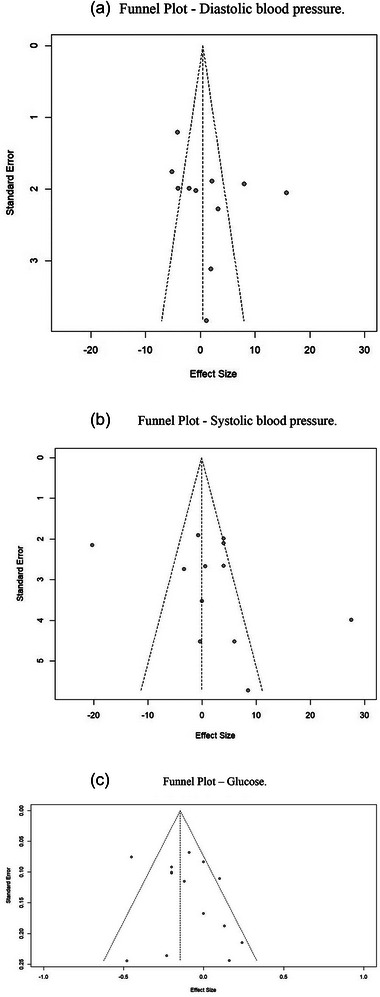
(a) Funnel plot for diastolic blood pressure. (b) Funnel plot for systolic blood pressure. (c) Funnel plot for glucose.

No publication bias was found for DBP, SBP or glucose (*P* > 0.05; Table 5; Figure 2).

**TABLE 5:** Publication bias of the included studies.

Outcome	*n*	*P*‐value	*z*	*b*	95% Confidence interval
Diastolic blood pressure	11	0.32	1.05	−7.32	−24.88 to 10.24
Systolic blood pressure	12	0.30	1.08	−9.90	−31.73 to 11.92
Glucose	15	0.26	1.17	−0.28	−0.54 to −0.02

Meta‐regression analyses showed that body weight was not a significant moderator among studies examining DBP, SBP or BMI for glucose (*P* > 0.05) (Table 6).

**TABLE 6:** Meta‐regression analyses based on body mass index/body weight difference, age difference, and proportion of females.

Outcome	β	SE	*z*	*P*‐value	95% Confidence interval	τ^2^	*I* ^2^ (%)	*H* ^2^	*R* ^2^ (%)
Mean body weight/body mass index difference									
Diastolic blood pressure	−0.22	0.30	−0.74	0.46	−0.82 to 0.37	40.16	96.5	28.43	0
Systolic blood pressure	0.38	3.64	0.10	0.92	−6.75 to 7.51	3.64	57.6	2.36	0
Glucose	−0.03	0.05	−0.53	0.59	−0.12 to 0.07	0.04	86.4	7.34	0
Mean age difference									
Diastolic blood pressure	0.34	1.14	0.30	0.77	−1.90 to 2.58	50.14	97.3	37.32	0
Systolic blood pressure	0.05	0.28	0.17	0.86	−0.50 to 0.60	118.38	97.6	42.21	0
Glucose	0.001	0.003	0.39	0.70	−0.004 to 0.01	0.03	84.4	6.42	0
Proportion of females									
Diastolic blood pressure	−0.03	0.06	−0.46	0.64	−0.15 to 0.09	38.70	96.0	25.07	0
Systolic blood pressure	−0.22	0.08	−2.63	0.01^a^	−0.38 to −0.06	66.97	96.0	24.82	37.4
Glucose	0.003	0.003	0.96	0.34	−0.003 to 0.01	0.03	83.5	6.05	0

^a^Indicates significance.

### 3.6 Risk of bias

In terms of cluster randomized controlled trials (RCTs), we found 12 studies with some overall concerns (Barbic et al., 2019; Blaber et al., 2023; Downs et al., 2020; Hajj‐Boutros et al., 2023; Krachtis et al., 2022; Mastrandrea et al., 2025; Stenger et al., 2012; Ward et al., 2020, 2024; Yang et al., 2014; Yao et al., 2012; Zwart et al., 2009). There were concerns regarding insufficient information on randomization and/or allocation concealment, and that some studies had high drop‐out rates and/or missing data (Table 7).

**TABLE 7:** Risk of bias assessment of parallel randomized controlled trials.

Study	D1	D2	D3	D4	D5	Overall
Amirova et al., 2020	Low	Low	Some concerns	Low	Low	Low
Barbic et al., 2019	Some concerns	Low	Some concerns	Some concerns	Low	Some concerns
Blaber et al., 2023	Low	Some concerns	Some concerns	Low	Low	Some concerns
Deutz et al., 2013	Low	Low	Low	Low	Low	Low
Downs et al., 2020	Some concerns	Low	Low	Some concerns	Low	Some concerns
Eggelbusch et al., 2024	Low	Low	Low	Low	Low	Low
Fuchs et al., 2024	Low	Low	Low	Low	Low	Low
Hajj‐Boutros et al., 2023	Some concerns	Low	Some concerns	Low	Low	Some concerns
Harder‐Lauridsen et al., 2017	Low	Low	Low	Low	Low	Low
Hoenemann et al., 2024	Low	Low	Low	Low	Low	Low
Jurdana et al., 2015	Low	Low	Low	Low	Low	Low
Krachtis et al., 2022	Some concerns	Low	Low	Some concerns	Low	Some concerns
Li et al., 2017	Some concerns	Low	Low	Low	Low	Low
Petrocelli et al., 2020	Some concerns	Low	Low	Low	Low	Low
Sadeghian et al., 2022	Low	Low	Some concerns	Low	Low	Low
Shi et al., 2014	Some concerns	Low	Low	Low	Low	Low
Stenger et al., 2012	Some concerns	Low	Low	Low	Some concerns	Some concerns
Ward et al., 2020	Some concerns	Low	Low	Low	Some concerns	Some concerns
Ward et al., 2024	Some concerns	Low	Low	Low	Some concerns	Some concerns
Yang et al., 2014	Some concerns	Low	Some concerns	Low	Low	Some concerns
Yao et al., 2012	Some concerns	Low	Low	Low	Low	Some concerns
Zwart et al., 2009	Some concerns	Low	Low	Low	Some concerns	Some concerns

Furthermore, one study (Heer et al., 2014) was scored as having some concerns overall owing to additional bias arising from measurement of the outcome of interest as part of the risk of bias assessment for crossover studies (Table 8).

**TABLE 8:** Risk of bias assessment of crossover randomized controlled trials.

Study	D1	D2	D3	D4	D5	Overall
Heer et al., 2014	Some concerns	Low	Low	Some concerns	Some concerns	Some concerns
Liphardt et al., 2018	Some concerns	Low	Low	Low	Low	Low
Mulder et al., 2014	Low	Low	Low	Low	Low	Low
Rudwill et al., 2018	Some concerns	Low	Low	Low	Low	Low
Trakaki et al., 2020	Low	Low	Low	Low	Low	Low

Finally, using ROBINS‐I for the non‐RCTs, only one study had an overall low risk of bias (Nosova et al., 2014). One study had a serious risk (Montero et al., 2018) owing to a lack of confounding factors being taken into account and the selection of reported results, and all others had a moderate risk of bias. Moderate risk was raised owing to confounders not being taken into account, missing data and/or measurement of the outcome of interest (Table 9).

**TABLE 9:** Risk of bias assessment of non‐randomized controlled trials.

Study	D1	D2	D3	D4	D5	D6	D7	Overall
Alibegovic et al., 2010	Moderate	Low	Low	Low	Low	Moderate	Moderate	Moderate
Coker et al., 2014	Moderate	Low	Low	Low	Low	Moderate	Low	Moderate
Convertino et al., 1990	Serious	Moderate	Low	Low	Moderate	Low	Low	Moderate
Højbjerre et al., 2010	Moderate	Low	Low	Low	Low	Moderate	Low	Moderate
Mikines et al., 1989	Moderate	Low	Low	Low	Moderate	Low	Low	Moderate
Montero et al., 2018	Serious	Moderate	Moderate	Low	Moderate	Moderate	Serious	Serious
Nosova et al., 2014	Moderate	Low	Low	Low	Low	Low	Low	Low
Shangraw et al., 1988	Moderate	Low	Low	Low	Moderate	Low	Low	Moderate
Shi et al., 2014	Moderate	Low	Low	Low	Moderate	Low	Low	Moderate
Smeuninx et al., 2021	Moderate	Low	Low	Low	Low	Moderate	Low	Moderate
Sonne et al., 2011	Moderate	Low	Low	Low	Moderate	Low	Low	Moderate
Yanagibori et al., 1998	Moderate	Low	Low	Low	Moderate	Low	Low	Moderate
Zorbas et al., 1992	Moderate	Low	Low	Low	Moderate	Low	Low	Moderate

## 4 DISCUSSION

This meta‐analysis elucidated the time course and magnitude of change in markers of cardiometabolic health in response to HBR and HDT. HBR of ≤14 days modestly reduced fasting glucose, whereas changes were non‐significant following shorter durations of HBR (≤7 days) or during any duration of HDT. Fasting insulin significantly increased with both HBR of ≤14 days and HDT of ≤60 days. HOMA‐IR significantly increased in response to ≤30 days of HBR and ≤60 days of HDT, indicative of increasing insulin resistance following periods of immobilization. Circulating blood lipids demonstrated distinctive responses to HDT and HBR. HDT of ≤60 days significantly reduced TC (MD, −13.89 mg/dL), and HBR in younger adults (≤30 days) significantly raised triglycerides (MD, 18.96 mg/dL). HDL‐C decreased significantly in response to both HBR and HDT (HBR of ≤14 days: MD, −4.53 mg/dL; and HDT of ≤60 days: MD, −6.89 mg/dL), with no heterogeneity in HDT (*I* ^2^ = 0%), pointing to a robust inactivity‐driven effect. Inflammatory markers (CRP and TNF‐a) showed significant increases following HBR of ≤14 days (CRP: MD, 1.57; TNF‐a: 1.33), whereas only TNF‐a was significantly elevated following ≤21 days HDT (MD: 0.40). Blood pressure showed no consistent changes, although these data were accompanied by high heterogeneity.

The significant rise in fasting insulin and HOMA‐IR with both HBR and HDT indicates a consistent peripheral insulin resistance during prolonged muscle disuse. Despite the decline in fasting glucose during the shorter‐term HBR (≤7 days), the increase in fasting insulin might suggest a hyperinsulinaemia response to maintain glucose homeostasis. This result from the analysis is concurrent with recent findings demonstrating that although skeletal muscle metabolism can recover rapidly from remobilization following inactivity, insulin resistance persists beyond the period of inactivity alone (Shur et al., 2024). The inclusion of studies in which HOMA‐IR was not directly reported and was derived from fasting glucose and fasting insulin data allowed for a more robust analysis and stronger findings by expanding the number of included studies. We were able to extract data for HOMA‐IR directly from only three studies; however, after the widely recognized formula to report HOMA‐IR (where it was not primarily reported) was used, we were able to power the analysis to *n* = 9 for HBR and *n* = 7 for HDT studies. The metabolic derangements driving declines in insulin sensitivity are likely to be linked to inactivity‐induced reduction in skeletal muscle mitochondrial oxidative capacity and/or intramyocellular lipid accumulation (Bilet et al., 2020). Additionally, a recent study of healthy young adults reported that 57 days of bed rest reduced glucose transporter‐4 (GLUT4) activity alongside noticeable impairments in mitochondrial function and poorer utilization of lipid‐derived fuels and increased intramuscular lipid accumulation (Eggelbusch et al., 2024). These alterations appear to act synergistically rather than independently, with reduced oxidative capacity amplifying the impact of diminished glucose transport, contributing to the inactivity‐driven insulin resistance. The significant increase in insulin in response of ≤60 days of HDT might suggest a compensatory response to gravitational unloading, possibly via increased pancreatic β‐cell secretion or reduced insulin clearance (Hall et al., 2023).

Considering the observed changes in circulating lipids, the responses differed between the bed rest models. Reductions in TC elicited by HDT, the increase in triglycerides following HBR and short‐term HDT might be attributable to decreased lipoprotein lipase activity in inactive muscle, favouring lipid storage (Zderic & Hamilton, 2006). Although a reduction in TC is typically observed as reducing the risk of cardiovascular disease, in the context of HDT, the effect we observe is likely to reflects the effect of the fluid shift altering the lipoprotein metabolism (Morvaridzadeh et al., 2024). The consistent decline in HDL‐C with both HBR and HDT might indicate reduced reverse cholesterol transport from diminished muscle HDL uptake. Impaired activities of peroxisome proliferator‐activated receptor delta (PPAR‐δ) and AMP‐activated protein kinase (AMPK) are known to drive a decline in muscle HDL‐C handling (Marques et al., 2018), although the impact of disuse on skeletal muscle HDL‐C uptake is not well described.

Although the majority of outcomes analysed in this systematic review were metabolic (i.e., lipid, inflammatory, glucose and insulin markers), their link to increased cardiometabolic disease risk and cardiovascular morbidity are widely established. We recognize that direct measures of cardiorespiratory fitness, such as maximal O_2_ uptake or heart rate, would be preferred; however, these are rarely available in the reports of eligible studies. Therefore, we focused on outcomes consistently available across both muscle disuse models (HDT and HBR) and outcomes that are strongly related to chronic risks of disease. As such, we consider the inclusion of these parameters within a cardiometabolic study justified. Moreover, although our analyses found no significant alterations in blood pressure, there was significant variability, especially during HDT exposure. HDT is recognized for simulating the cephalad fluid shifts typically observed in spaceflight, and consequently, variations in blood pressure would be anticipated owing to shifts in cardiac blood volume, autonomic regulation and baroreceptor responsiveness (Blaber et al., 2023). This could be a compensatory mechanism to preserve mean arterial pressure, despite the shift in fluid distribution, which has been seen in orthostatic intolerance studies (Ried‐Larsen et al., 2017).

Both models of inactivity also showed variable effects on markers of systemic inflammation (serum CRP, IL‐6 and TNF‐a), with limited overall impact on the analyses but high heterogeneity. This is likely to be reflective of variance in sampling times, assay sensitivity, low power and characteristics of participants at baseline levels. Although inflammation is more widely reported in animal models of disuse/immobilization, it is usually localized to the skeletal muscle (Hirata et al., 2022; Oura et al., 2025). Short‐term immobilization has been shown to alter perfusion and vascular function, which could explain changes in glucose uptake and more rapid increases in inflammation. Although HDT induces a cephalad fluid shift (which is key in the use of the model in spaceflight analogue research), the limited assessment of inflammation‐related markers within individual HDT studies means that no direct conclusions can be drawn about whether HDT attenuates or alters systemic inflammation. Further work in human models of disuse is needed to gain a better understanding of the factors influencing local inflammation and how they might influence skeletal muscle energetics.

### 4.1 Strengths and limitations

Although a number of outcomes from this meta‐analysis displayed low heterogeneity in key findings (e.g., *I* ^2^ = 0% for HDL‐C and insulin under HDT), many others exhibited moderate or substantial variability across studies (e.g., *I* ^2 ^> 60% for HOMA‐IR, triglycerides, SBP, DBP, CRP, IL‐6 and TNF‐a), which is likely to reflect the differences in study design, duration of exposure to the HBR model, participant baseline characteristics and measurement protocols. The absence of publication bias for glucose, SBP and DBP, in addition to the non‐significant BMI/body weight moderation via meta‐regression, enhances robustness for those specific outcomes.

This study, however, has several limitations. Importantly, high heterogeneity in some analyses (e.g., *I* ^2^ = 93%–95% for SBP under HDT or 96% for IL‐6 under HBR) reflects diverse study designs, durations and populations. Resting heart rate, which is commonly reported as a marker of cardiorespiratory fitness, was rarely available across the eligible studies, preventing quantitative analysis. Future studies including direct measures of cardiorespiratory fitness would better clarify whether any metabolic changes are associated with alterations in cardiovascular function or whether they are reflective of disruption in lipid handling or insulin resistance. A further limitation associated with the present analysis relates to inconsistencies in the terminology and/or reporting of body composition outcomes across the studies. In several reports, values described as ‘lean mass’ appeared to be representative of fat‐free mass. To maintain methodological consistency and avoid any subjectivity in our outcomes, we excluded the use of lean mass and/or fat‐free mass from our analyses. This decision was made to ensure body composition changes truly reflected skeletal muscle‐related adaptations rather than mixed tissue‐type analyses, which would be likely to affect the results and conclusions of this study. Although sex is a biologically relevant moderator, inconsistent reporting and a marked sex imbalance across studies meant that statistically meaningful analysis of sex as a moderator would be underpowered. Furthermore, small sample sizes for certain outcomes (e.g., *n* = 3 for HDT regarding CRP and TNF‐a) limit statistical power, and some studies had an increasing risk of bias (11 RCTs with ‘some concerns’ and one non‐RCT with serious risk).

Moreover, this meta‐analysis used within‐group rather than between‐group pre‐to‐post intervention differences, considering the lack of a control group. Finally, cross‐sectional data preclude causality, and unexamined confounders in the included studies (e.g., sex, nutrient intake, baseline physical activity) might not allow the extrapolation of insights on their impact, warranting their incorporation in future research.

## 5 CONCLUSION

In conclusion, both HBR and HDT altered markers of cardiometabolic health in a time‐specific and disuse model‐specific manner. These changes were characterized by glucose clearance challenges, elevated insulin and HOMA‐IR, dyslipidaemia and elevated inflammatory responses. Blood pressure remained largely unchanged. However, because only SBP and DBP were readily reported across studies, and owing to the variability in the duration of exposure, the present data more clearly delineate disturbances in metabolism rather than haemodynamic disturbances. Additional work is needed to tease out the age‐specific burden of disuse on cardiometabolic health, especially given that older adults are more likely to experience enforced situations of disuse (i.e., hospital stay). Despite methodological heterogeneity and bias risks, this meta‐analysis indicates a need for longitudinal and intervention studies (e.g., nutrition and contractile activity) to gain a better understanding of the impact of, and potential mitigation strategies for, the decline in insulin sensitivity and altered blood lipid profiles in populations affected by HBR and/or HDT.

## AUTHOR CONTRIBUTIONS

Konstantinos Prokopidis designed the study. Konstantinos Prokopidis and Tyler Daubrah‐Scott performed the data extraction. Konstantinos Prokopidis, Tyler Daubrah‐Scott, and Jordi Morwani‐Mangnani worked on the risk of bias assessment. Konstantinos Prokopidis conducted the analysis. Konstantinos Prokopidis, Tyler Daubrah‐Scott, Alyssa Varanoske, and Emily J. Arentson‐Lantz wrote the manuscript. Alyssa Varanoske, David D. Church, Colleen S. Deane, Bethan E. Phillips, and Emily J. Arentson‐Lantz revised the manuscript.

## CONFLICT OF INTEREST

Emily J. Arentson‐Lantz has received honoraria and travel reimbursement from the National Pork Board, the Beef Checkoff Program managed by National Cattlemen's Beef Association (NCBA), and the US Dairy Export Council. David D. Church is currently supported by a National Institutes of Health (NIH) Clinical Research Loan Repayment Award. The content is solely the responsibility of the authors and does not necessarily represent the official views of the National Institutes of Health. The findings are presented clearly, honestly, and without fabrication, falsification, or inappropriate data manipulation. David D. Church has received research funding to conduct from the National Pork Board, Beef Checkoff Program managed by the NCBA, National Dairy Council. David D. Church has performed freelance work for Soy Connection funded by US Soy. David D. Church is on the advisory board for Shifted Supplements. David D. Church has received travel reimbursement from Protein PACT.

## FUNDING INFORMATION

None.

## Supporting information



Supporting Information

Supporting Information

## Data Availability

Data are available upon request.
