# Supplementary material for: The effects of bed rest on cardiometabolic health: A systematic review and meta‐analysis
Source: Exp Physiol. 2026 Mar 19:10.1113/EP092944. Online ahead of print. doi: 10.1113/EP092944 (PMC13394784; doi:10.1113/EP092944)
Supplement: Supplementary file 1 — Supporting Information [file EPH-9999-0-s002.docx]

**Supplementary File 1.** Forest plots of each outcome.

**Glucose (mmol/l)**

Bed rest (up to 7 days)

**
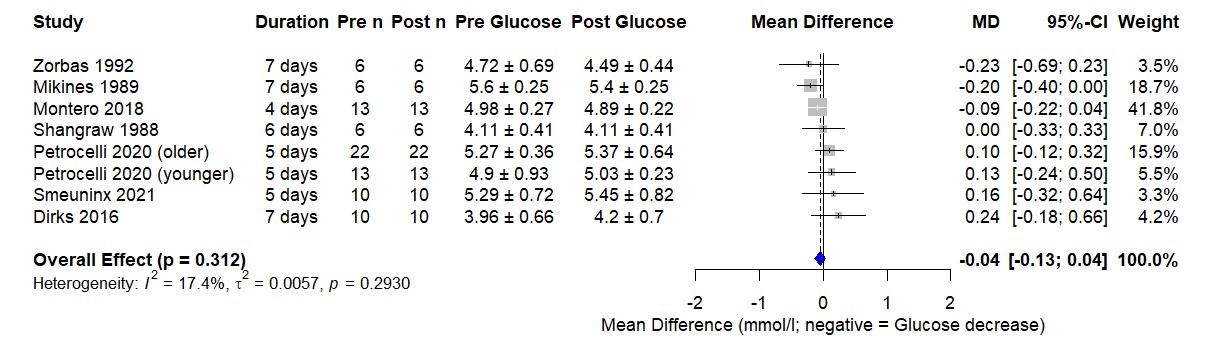
**

Bed rest (up to 14 days)


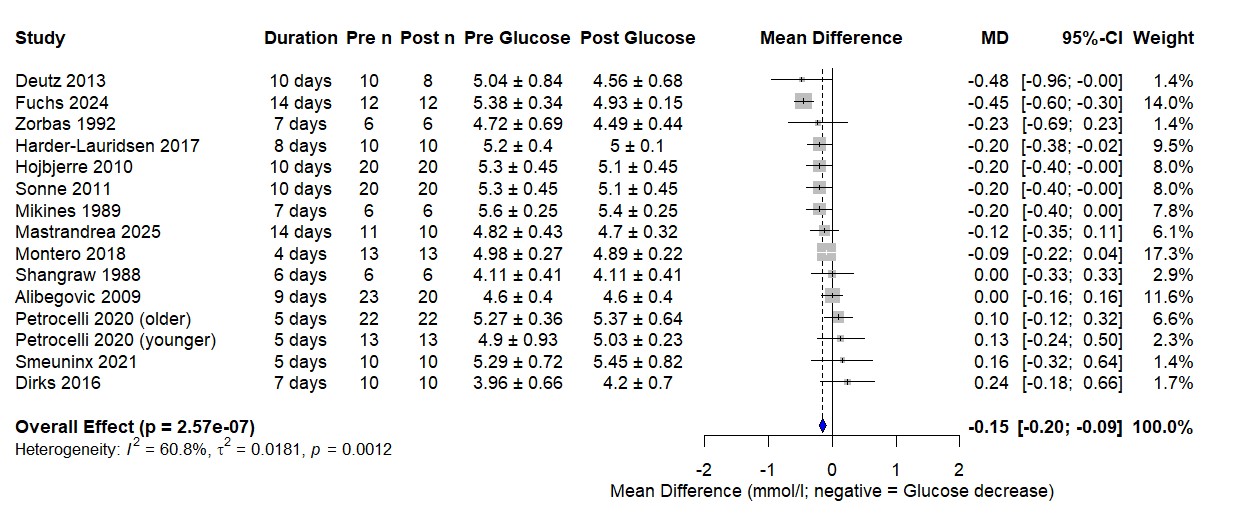


Bed rest (up to 14 days) – Including data with bodyweight changes


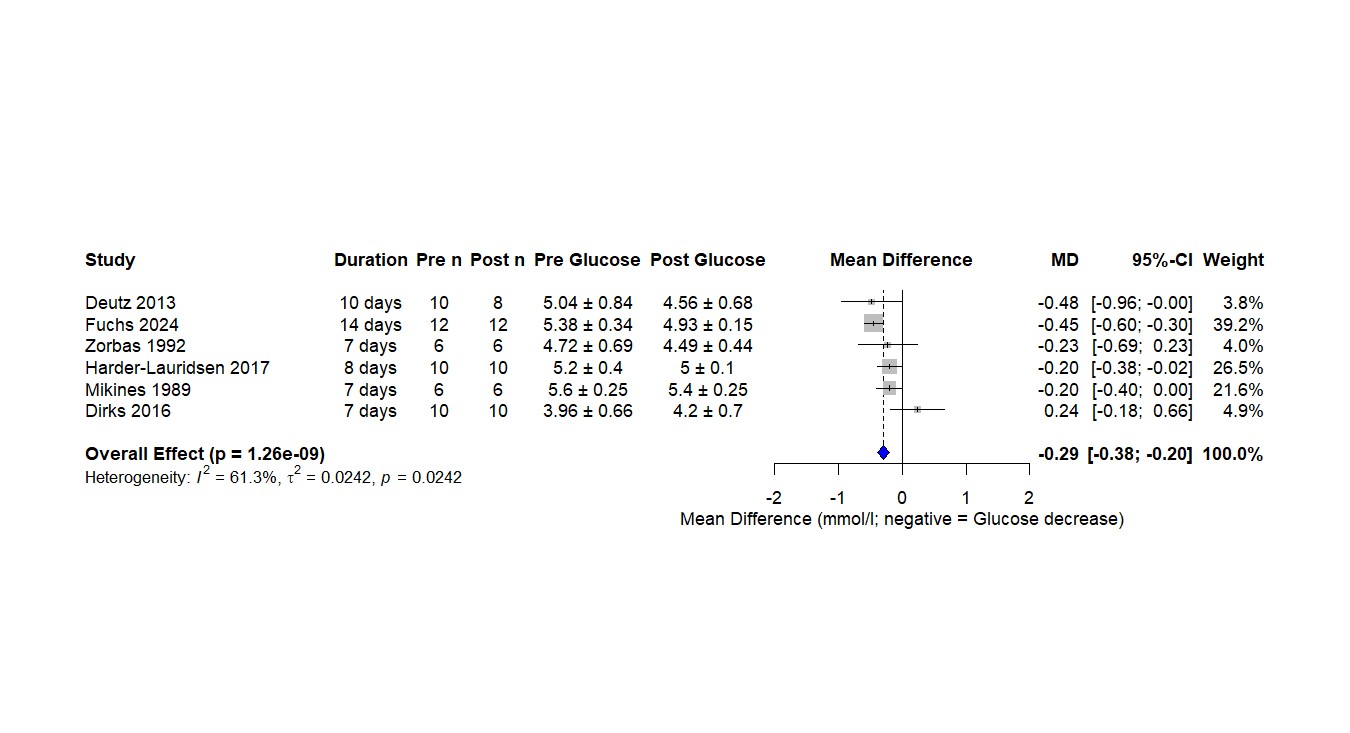


Head-down tilt (up to 21 days)


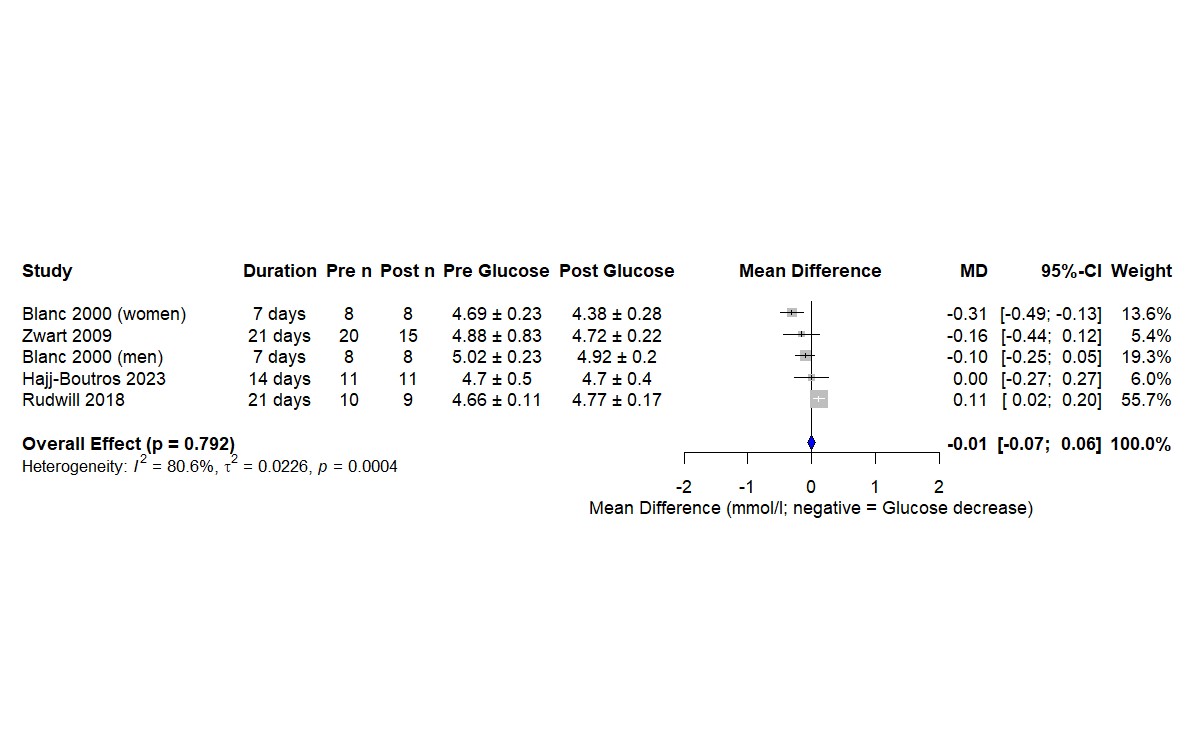


Head-down tilt (up to 60 days)


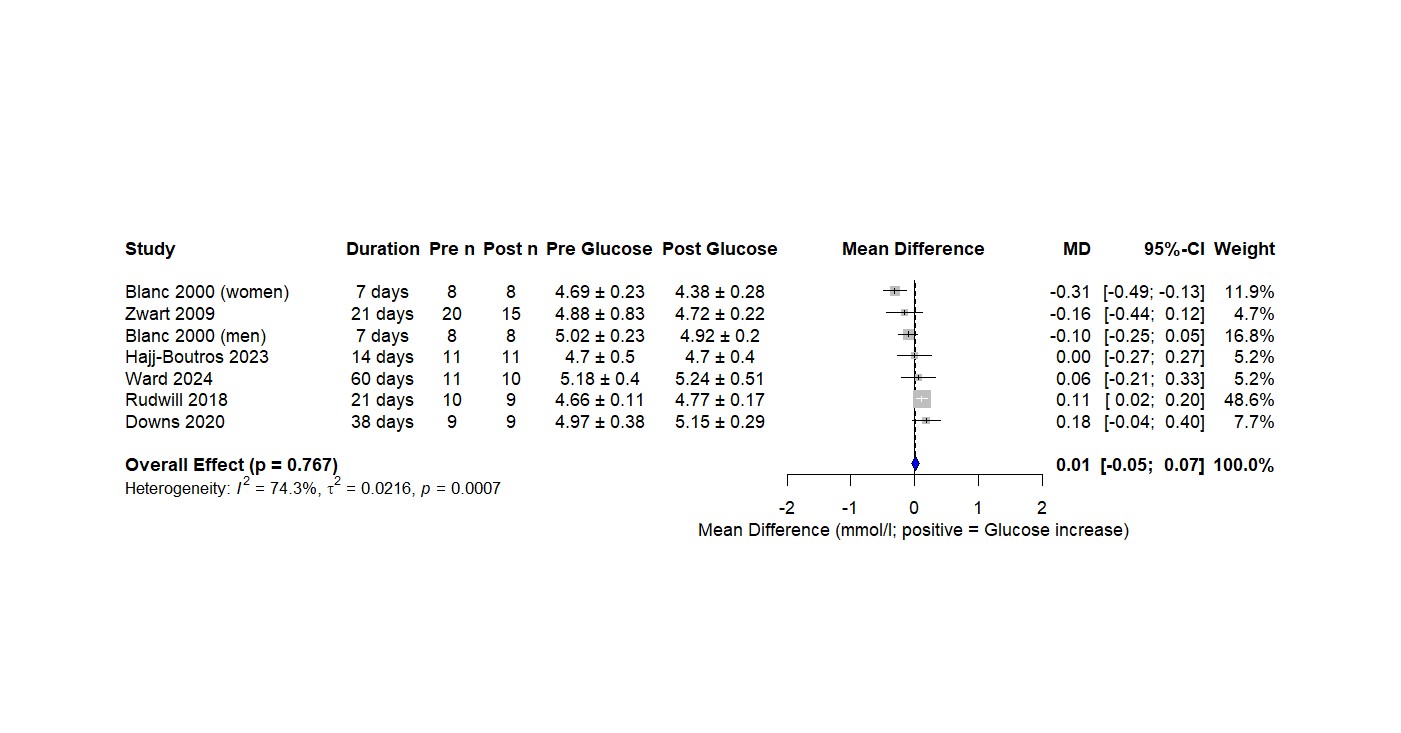


**Bodyweight (kg)** – Bed rest (up to 14 days)


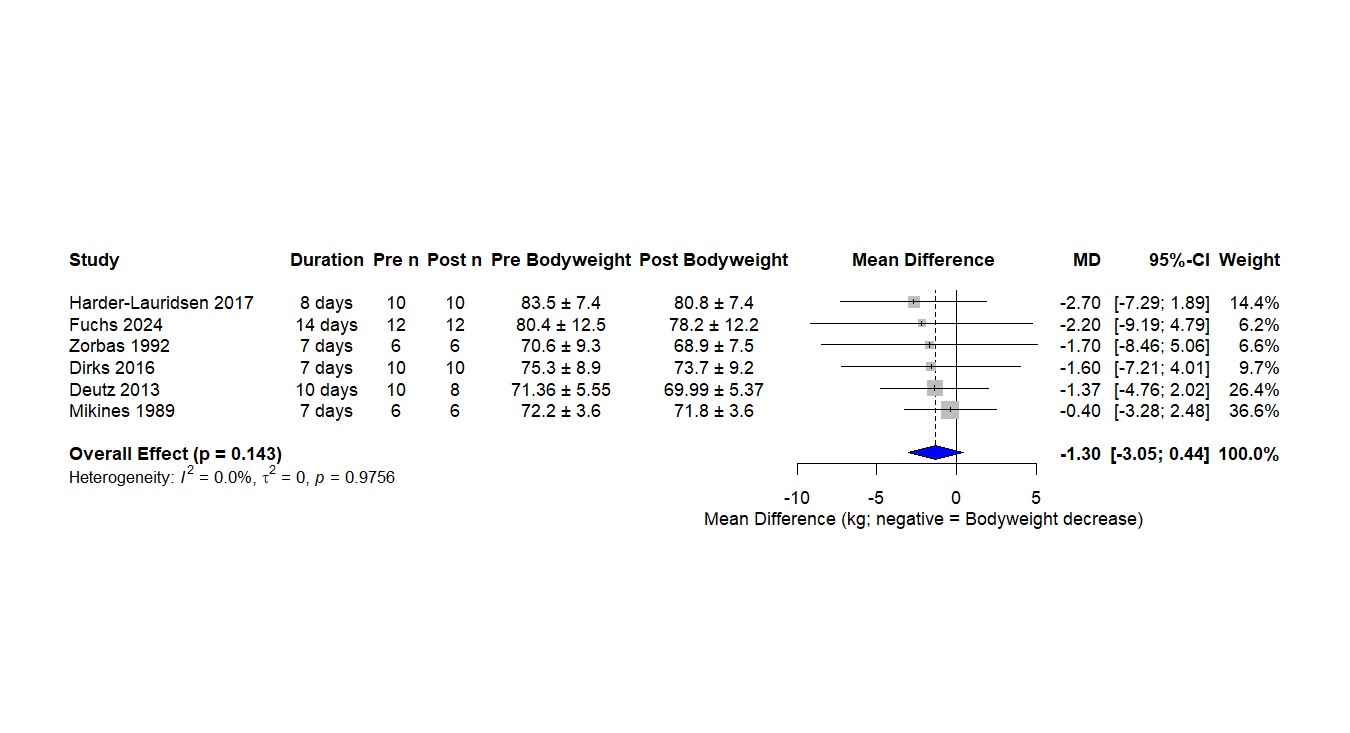


**Insulin (pmol/L)**

Bed rest (up to 14 days)


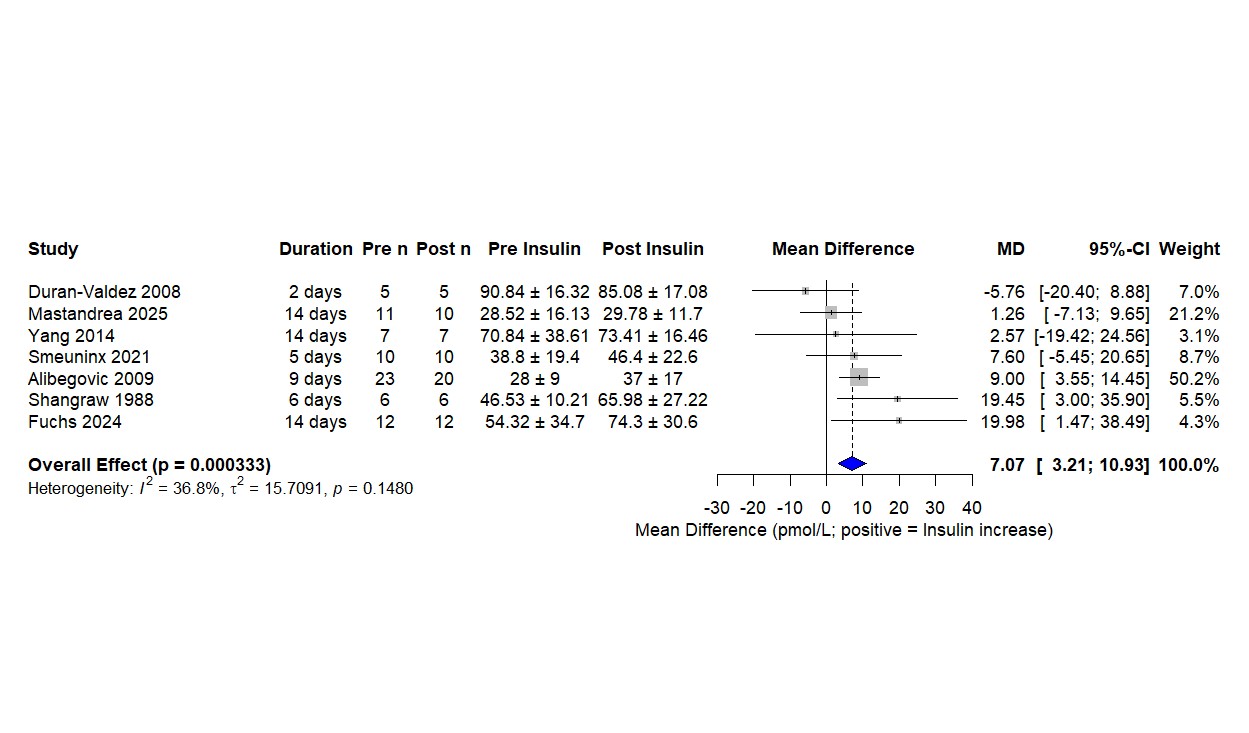


Head-down tilt (up to 21 days)


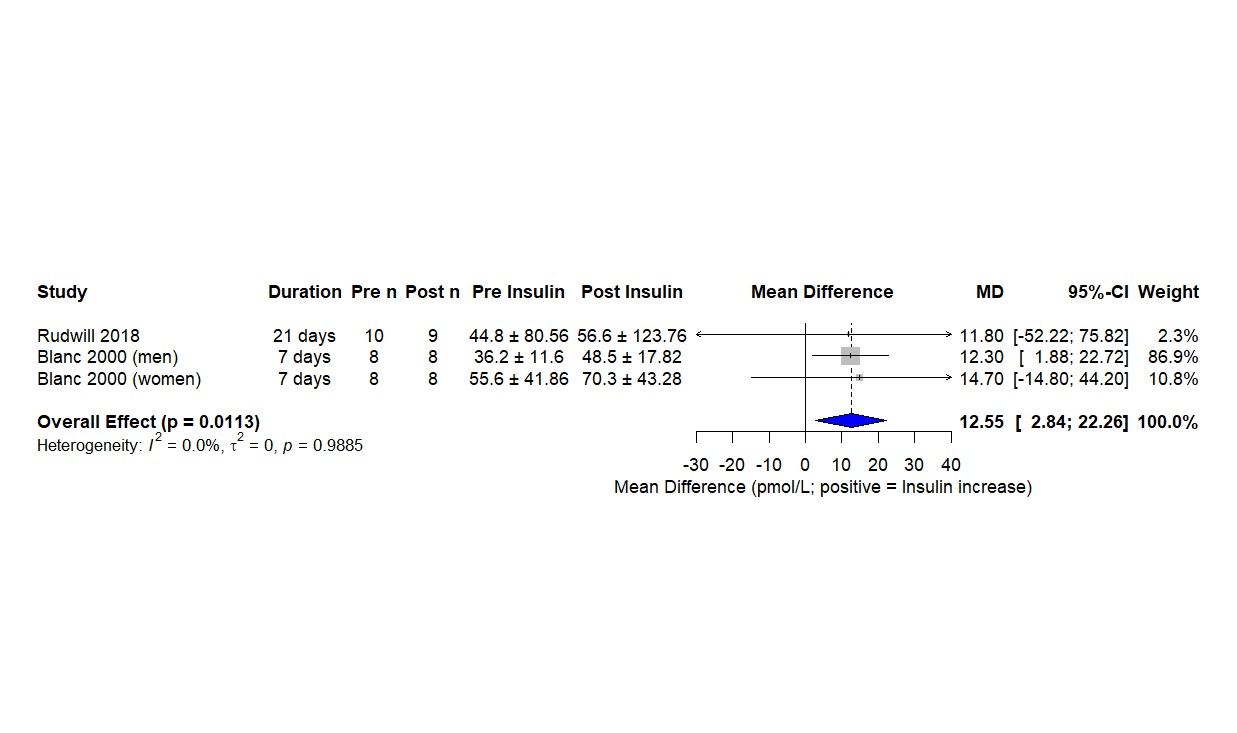


Head-down tilt (up to 60 days)


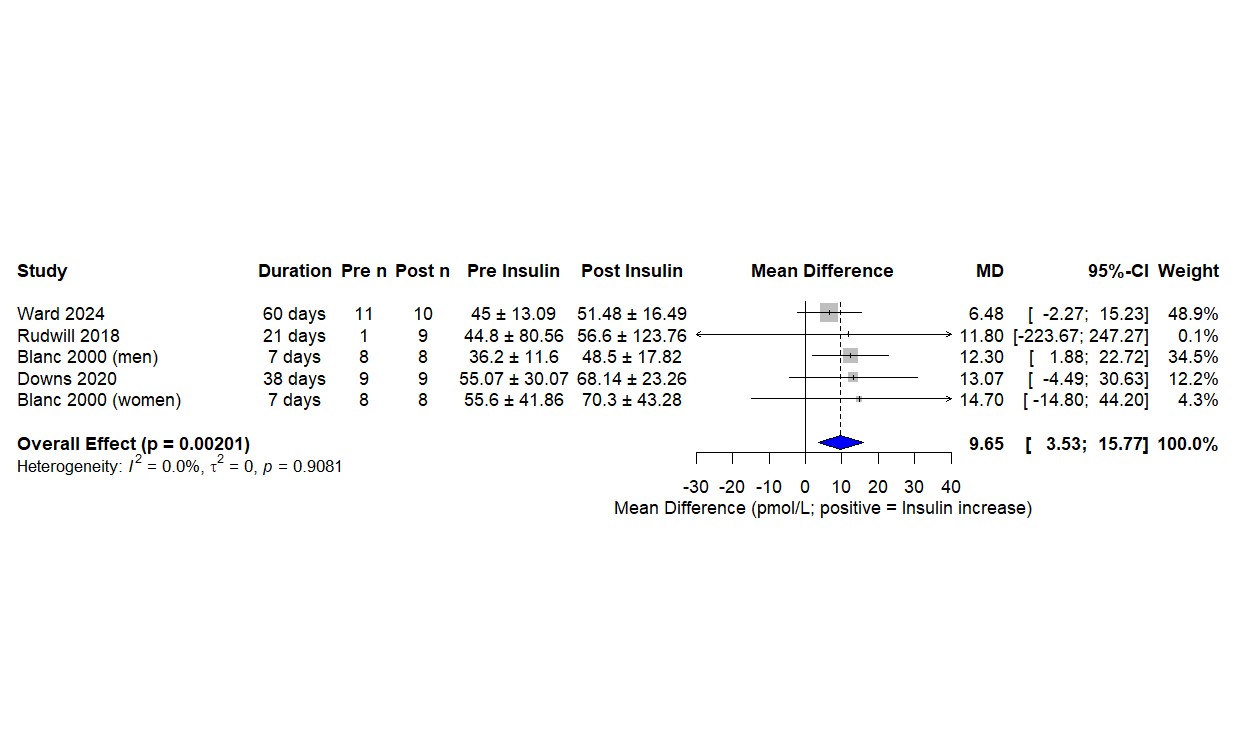


**HOMA-IR**

Bed rest (up to 14 days)


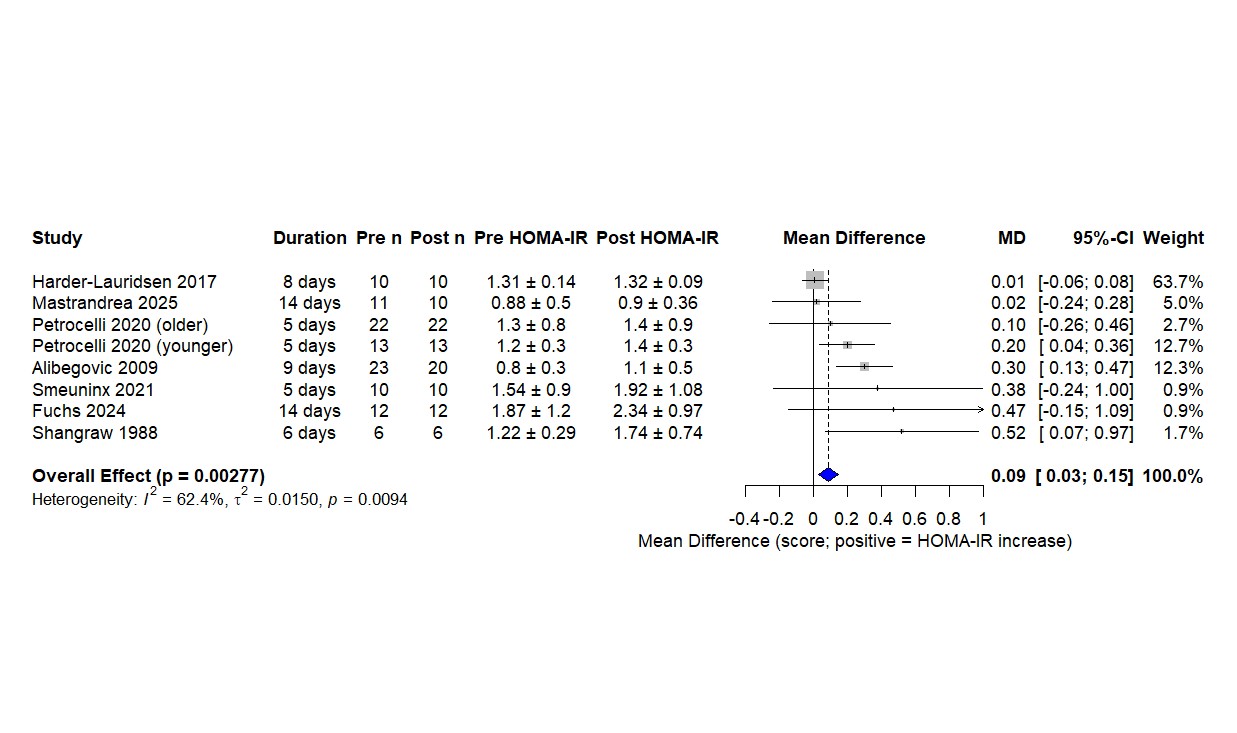


Bed rest (up to 30 days)


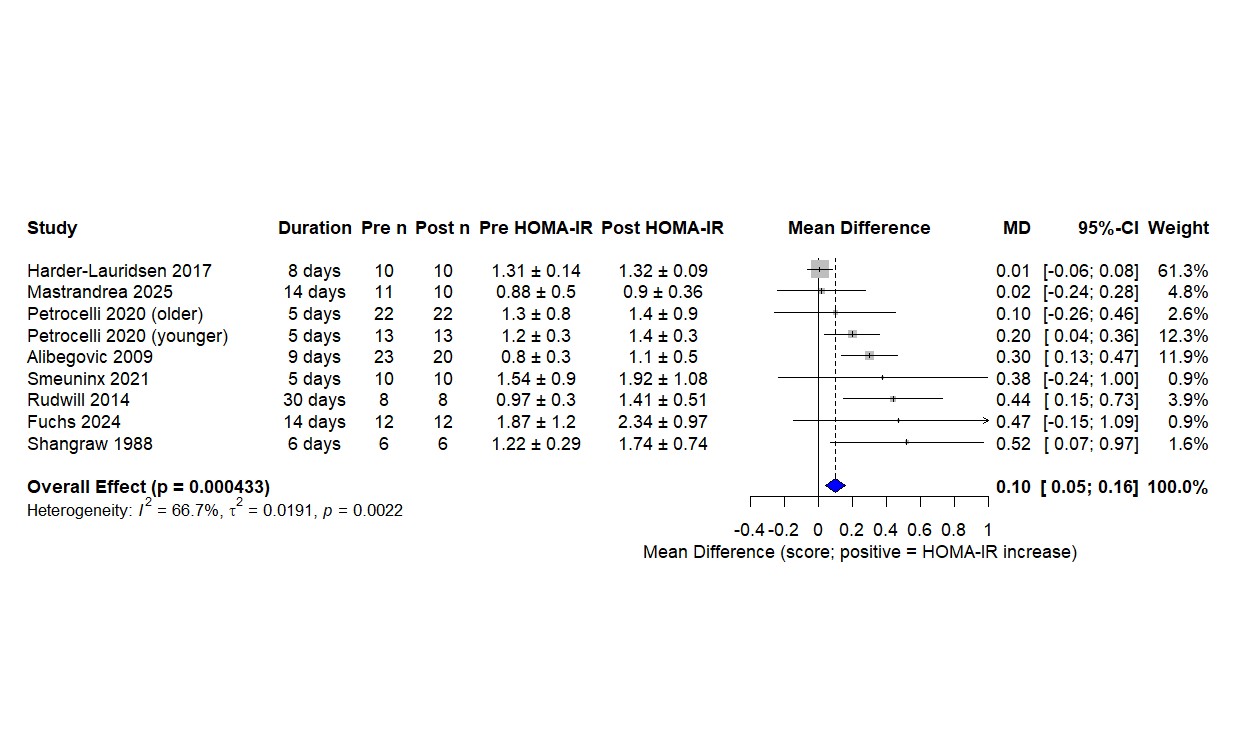


Bed rest (up to 30 days) – Including data with body mass index changes


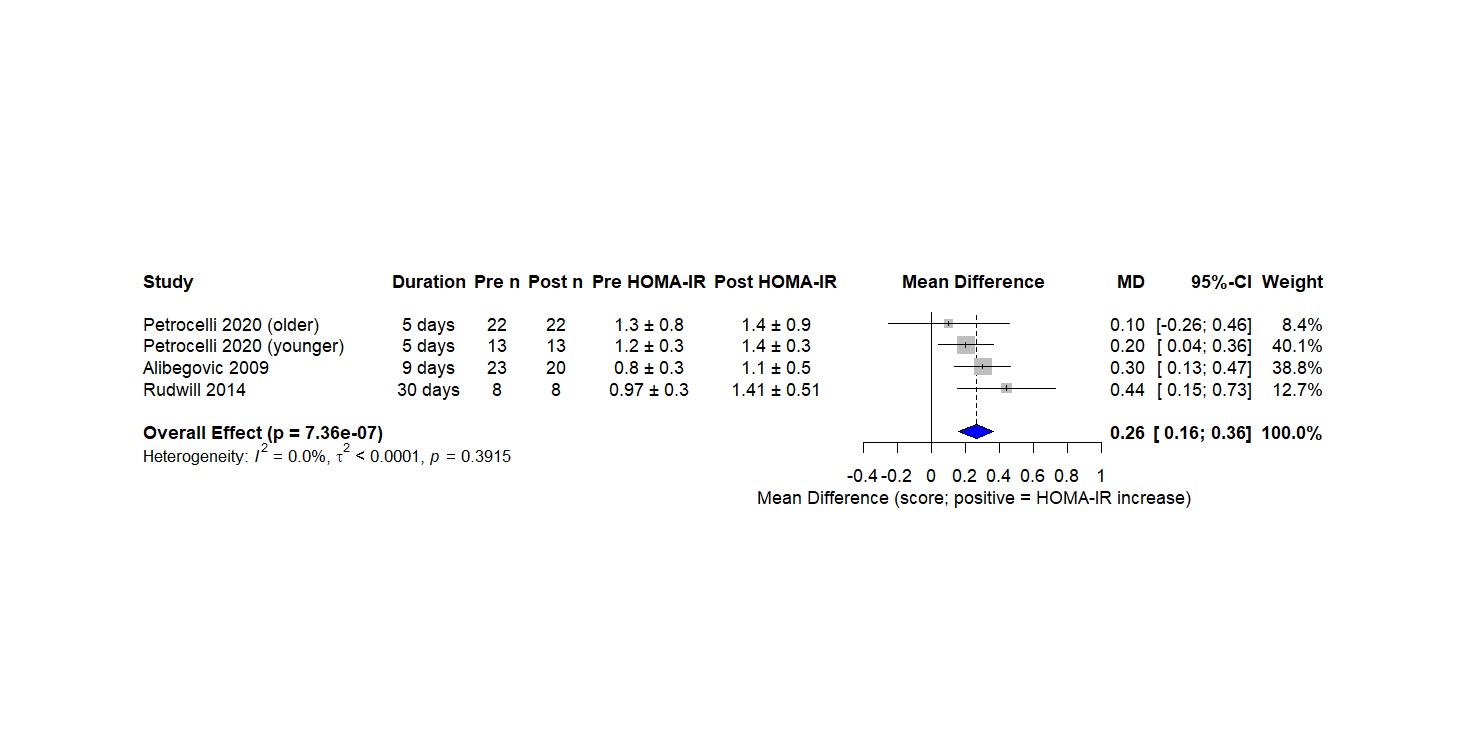


Head-down tilt (up to 21 days)


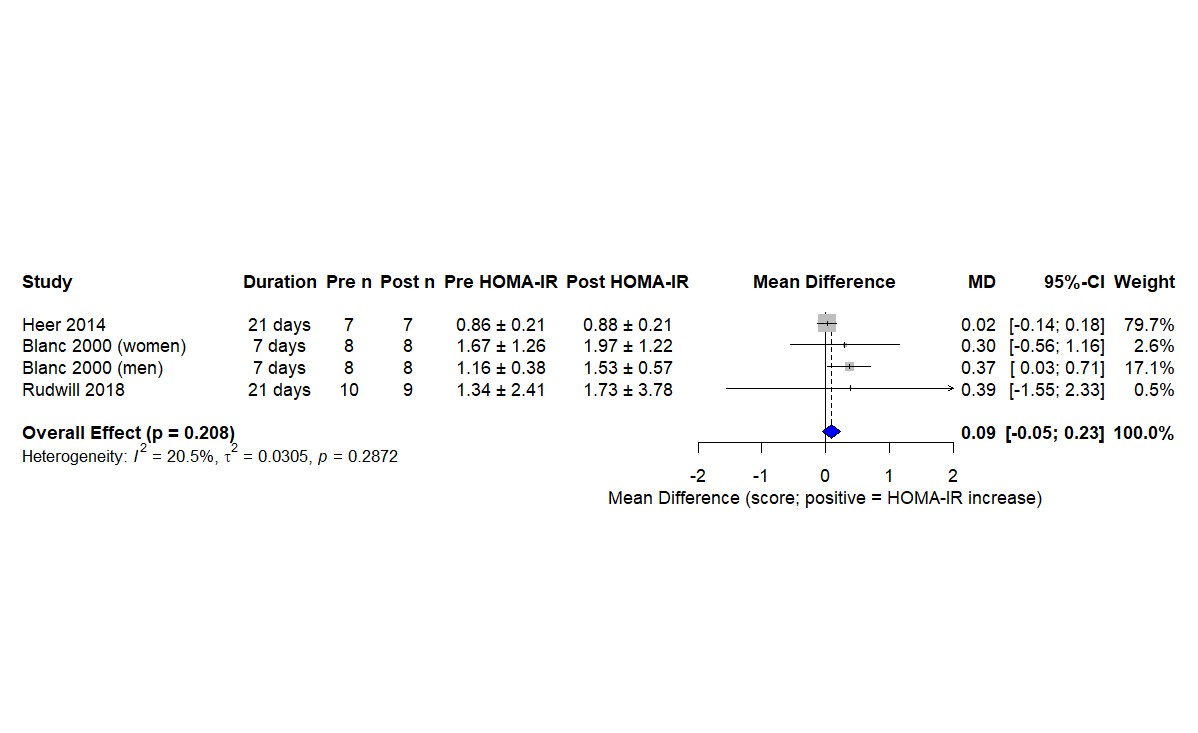


Head-down tilt (up to 60 days)


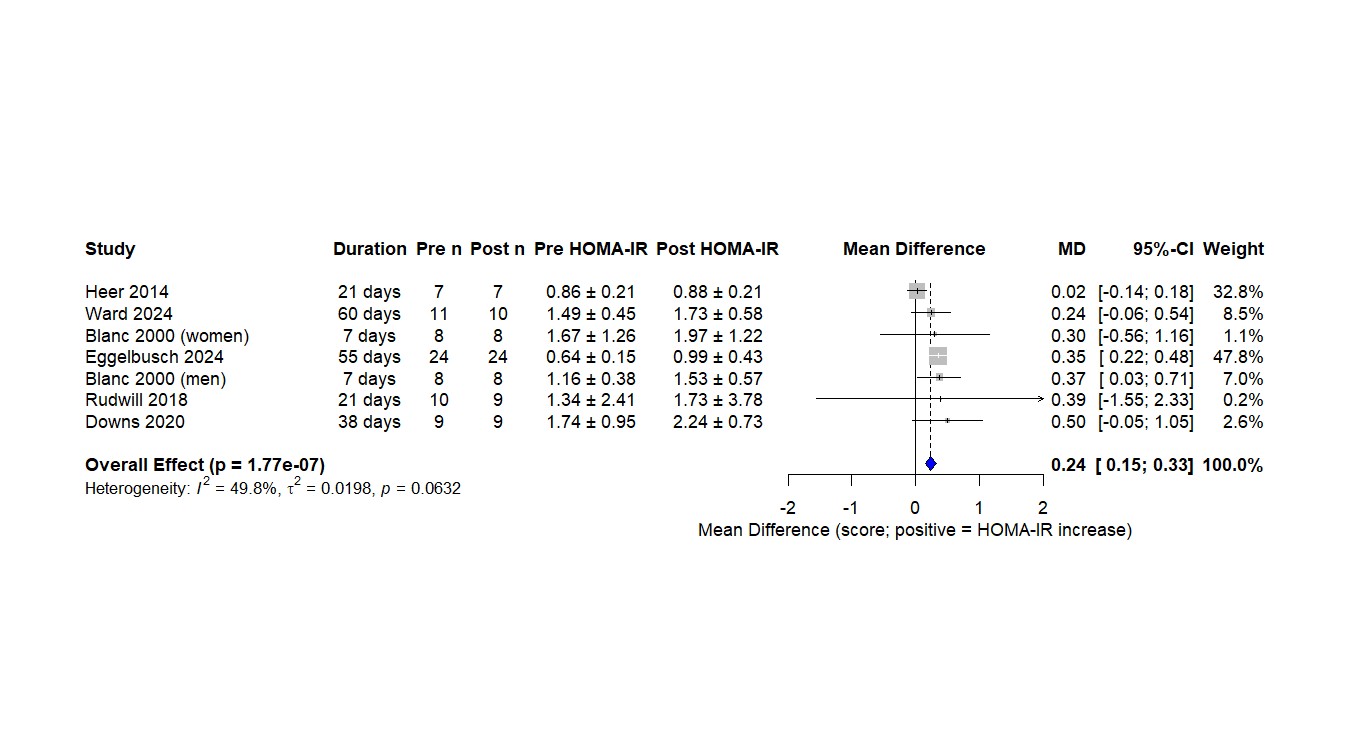


**Total cholesterol (mg/dL)**

Bed rest (up to 10 days)


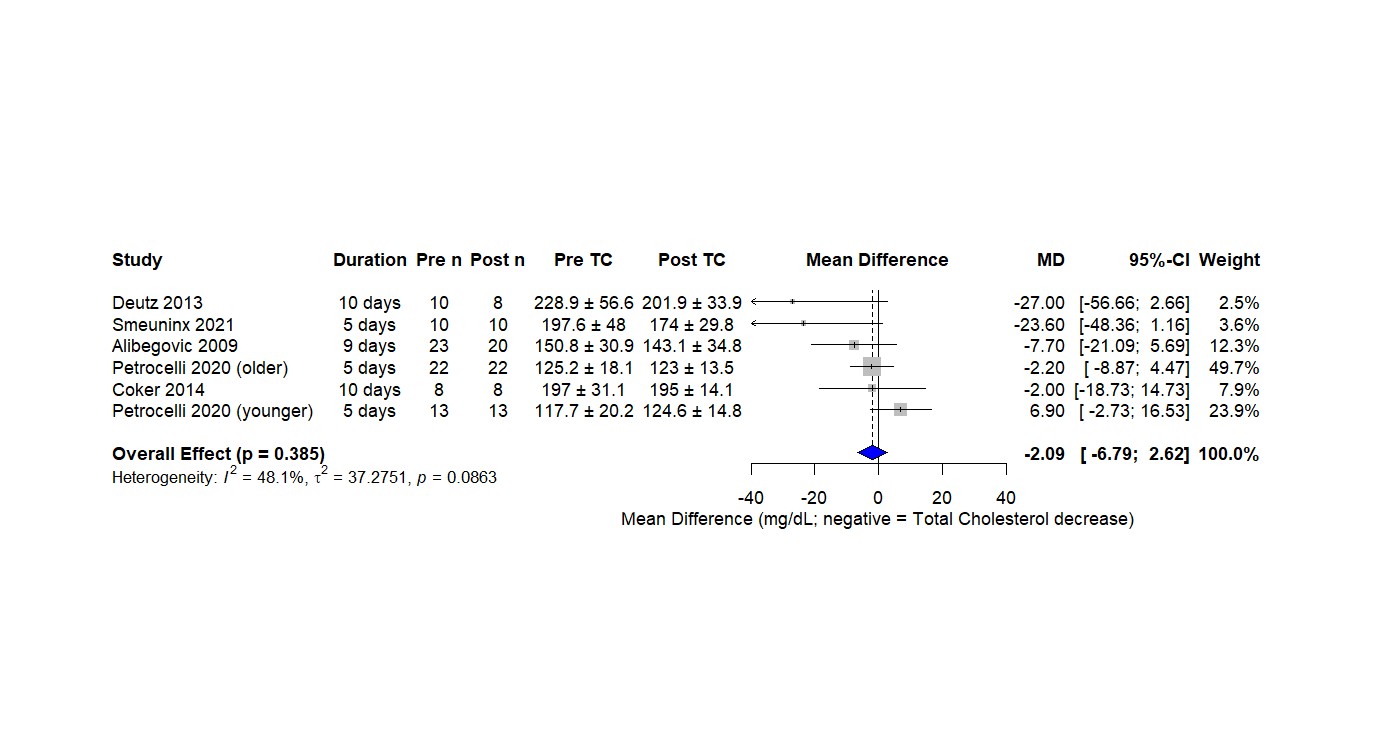


Head-down tilt (up to 21 days)


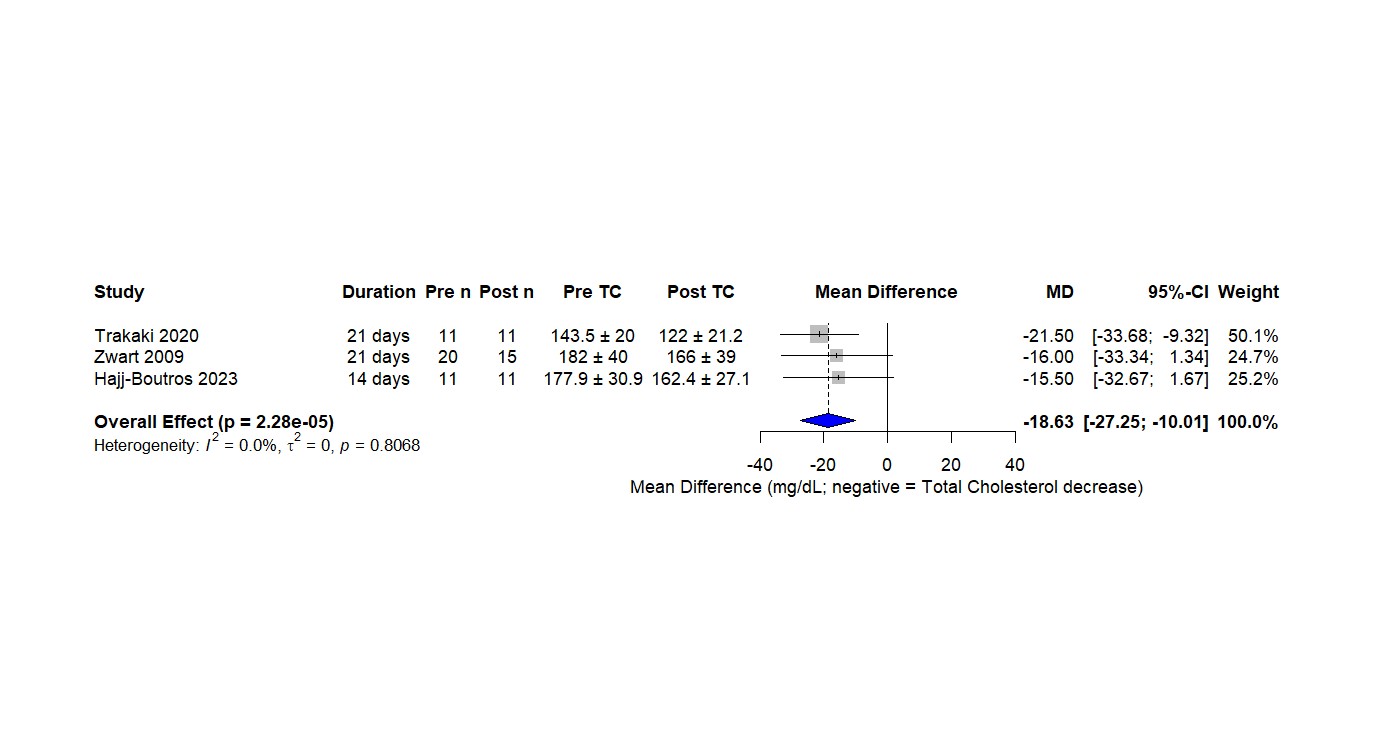


Head-down tilt (up to 60 days)


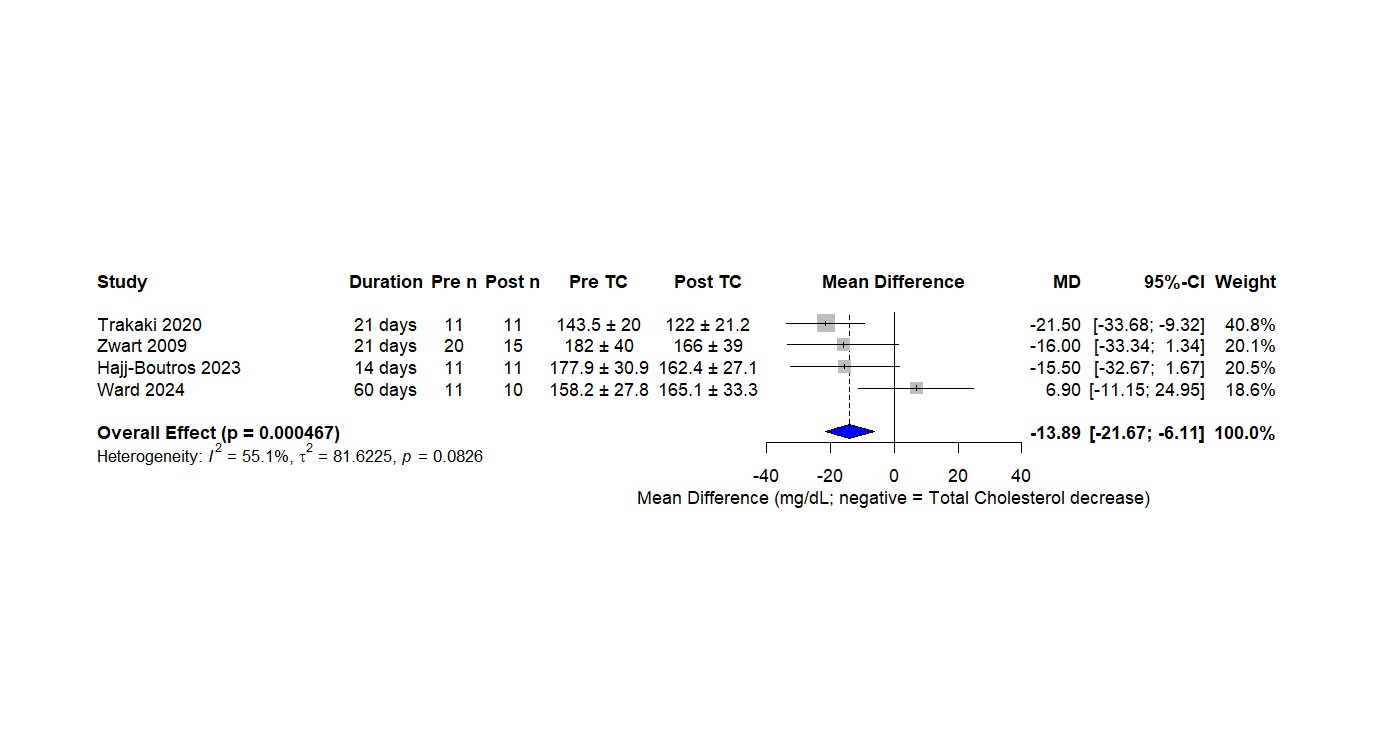


**Triglycerides (mg/dL)**

Bed rest (up to 10 days)

**
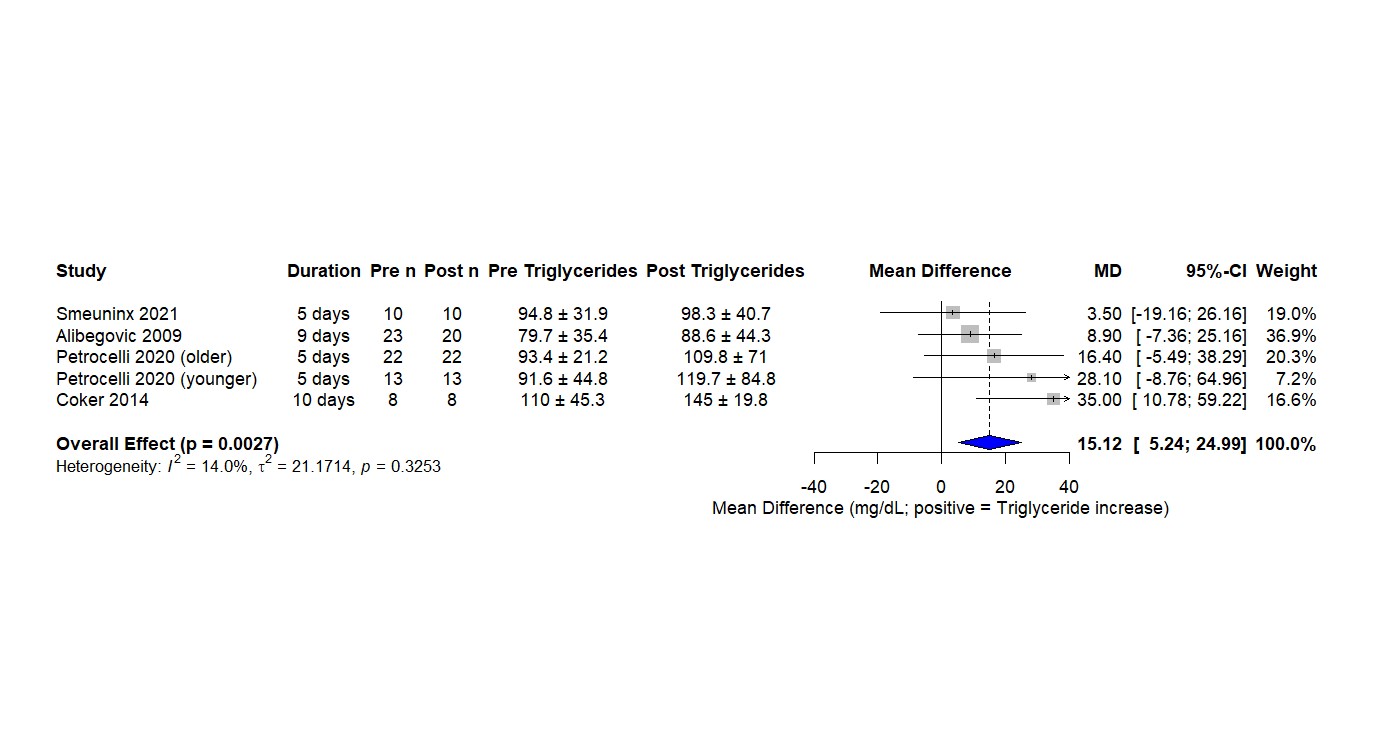
**

Bed rest (up to 20 days)

**
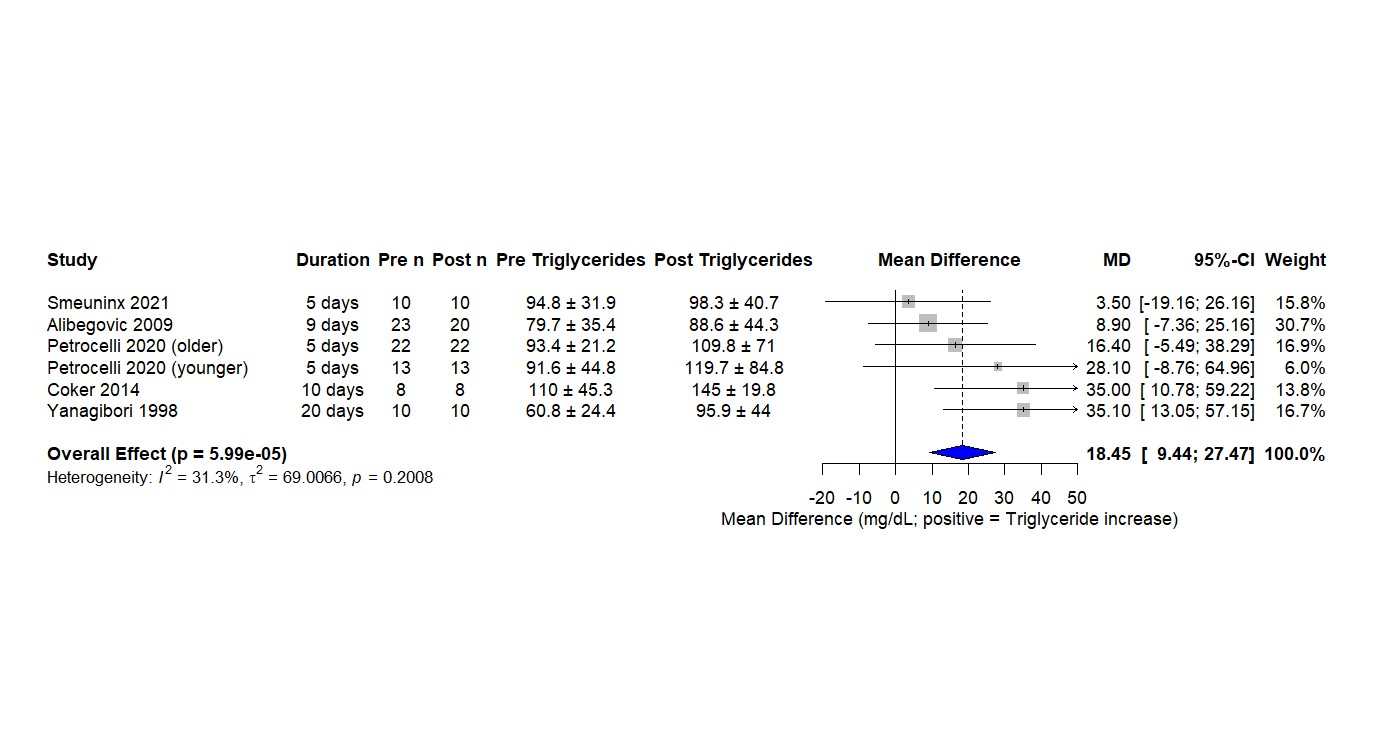
**

Bed rest (up to 30 days)

**
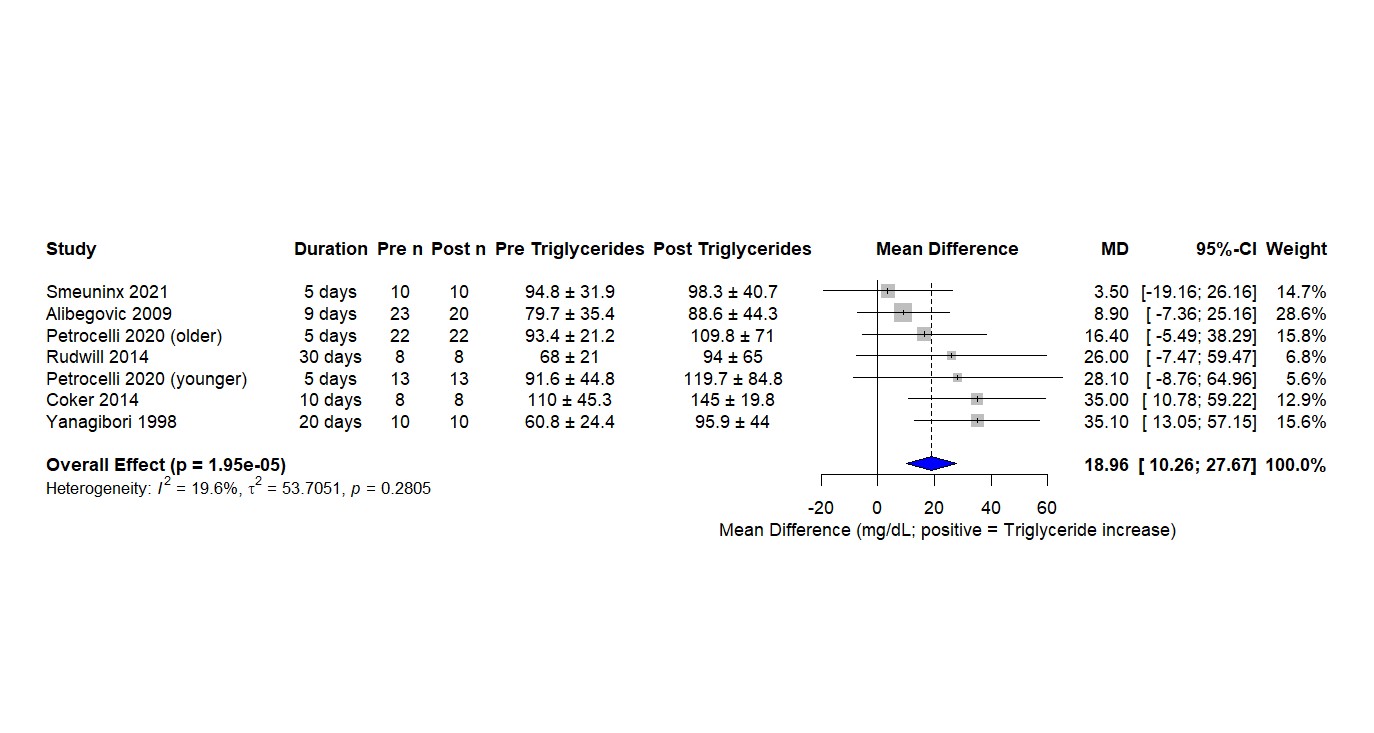
**

Bed rest (up to 30 days) – Including data with body mass index changes

**
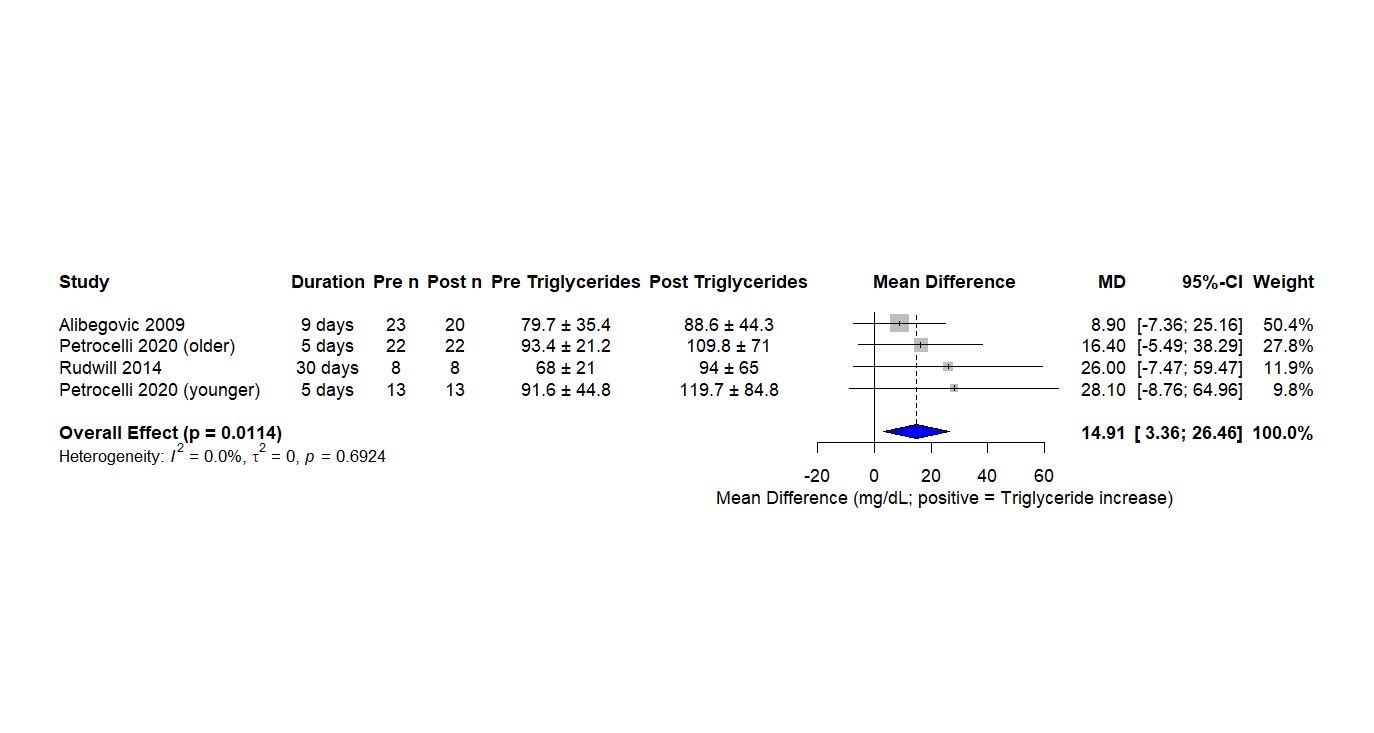
**

Head-down tilt (up to 21 days)

**
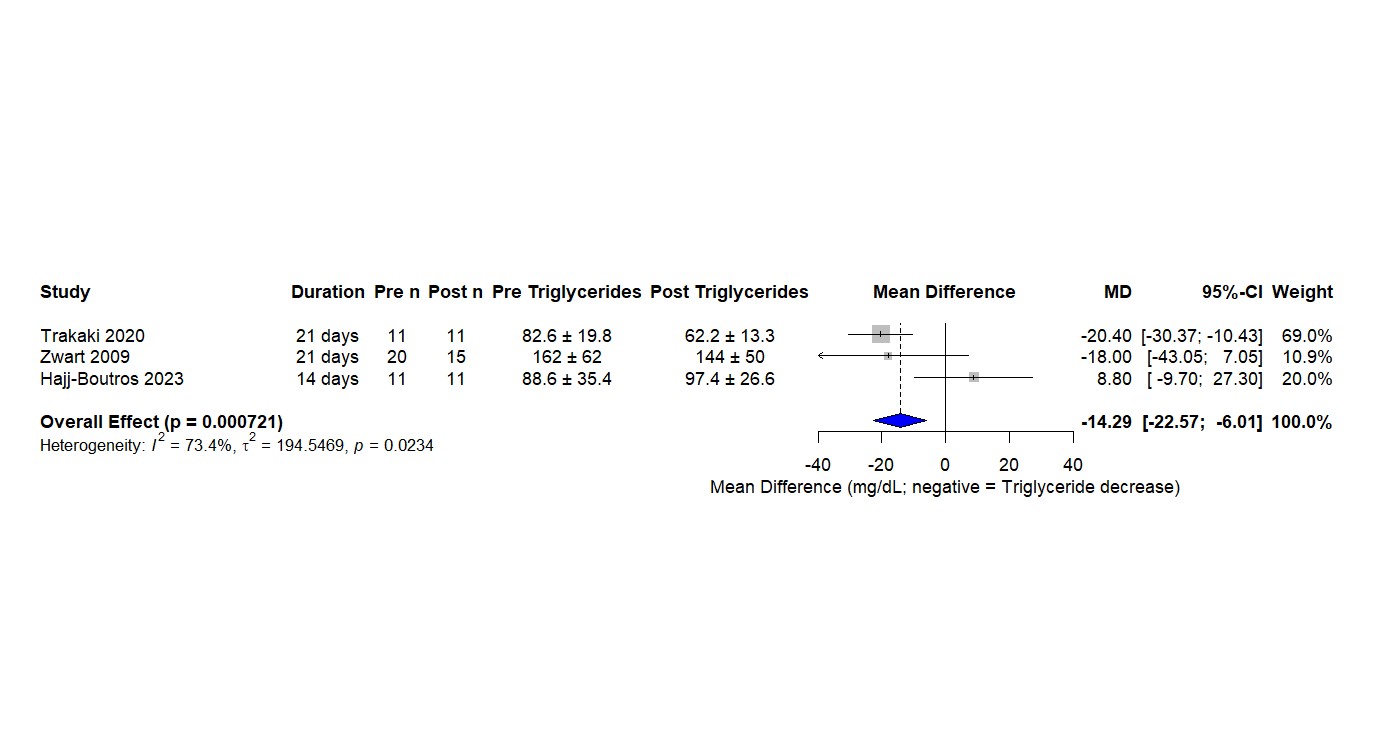
**

Head-down tilt (up to 60 days)


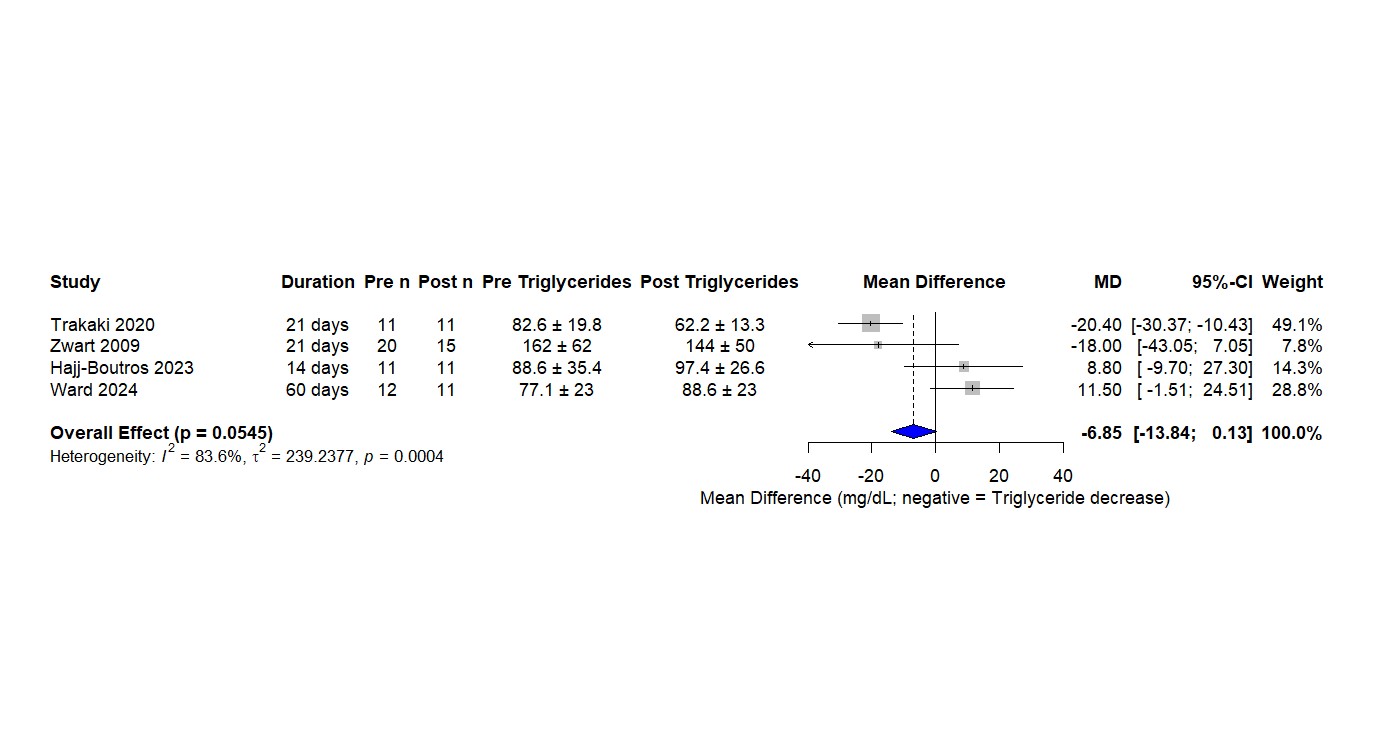


**High-density lipoprotein (mg/dL)**

Bed rest (up to 14 days)


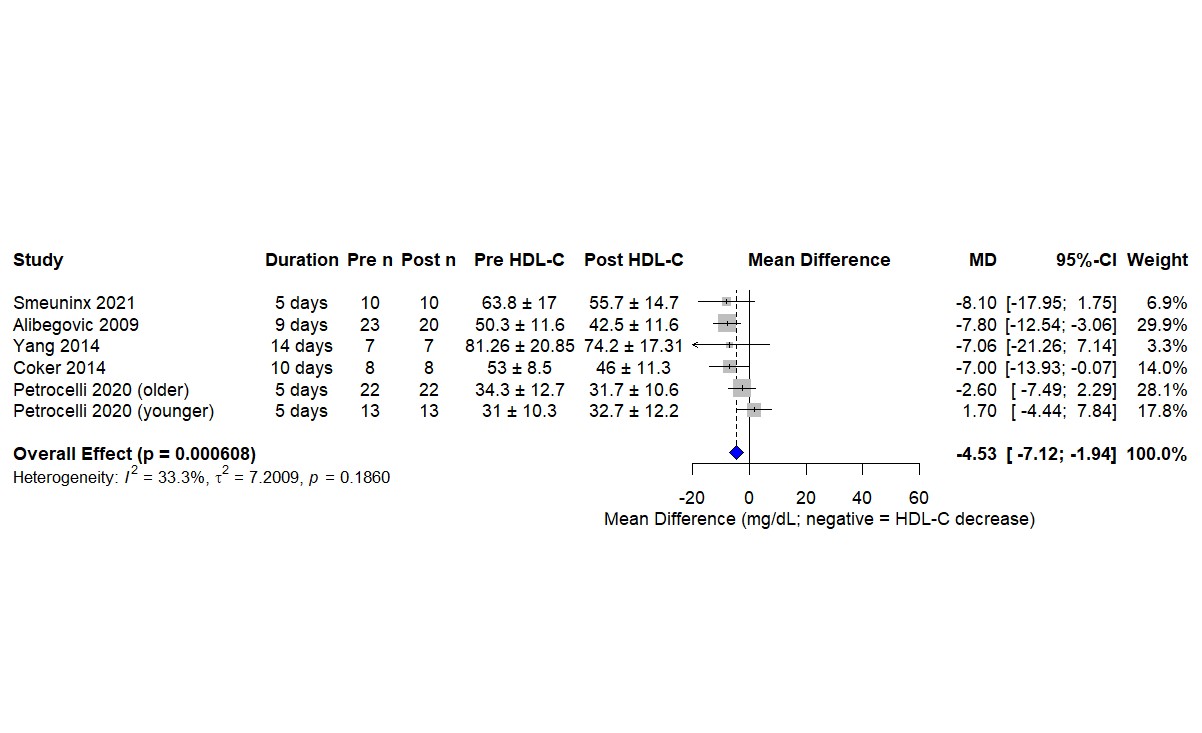


Head-down tilt (up to 60 days)


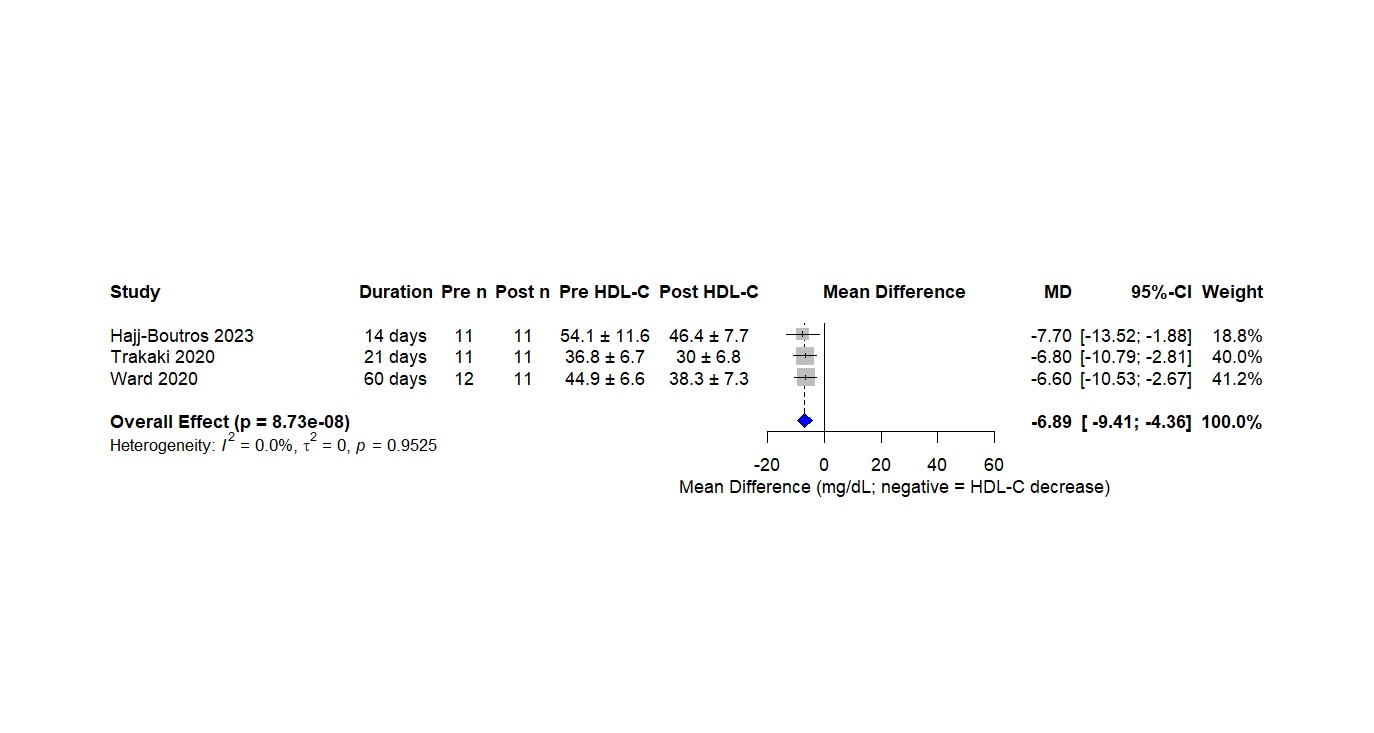


**Low-density lipoprotein (mg/dL)**

Bed rest (up to 14 days)


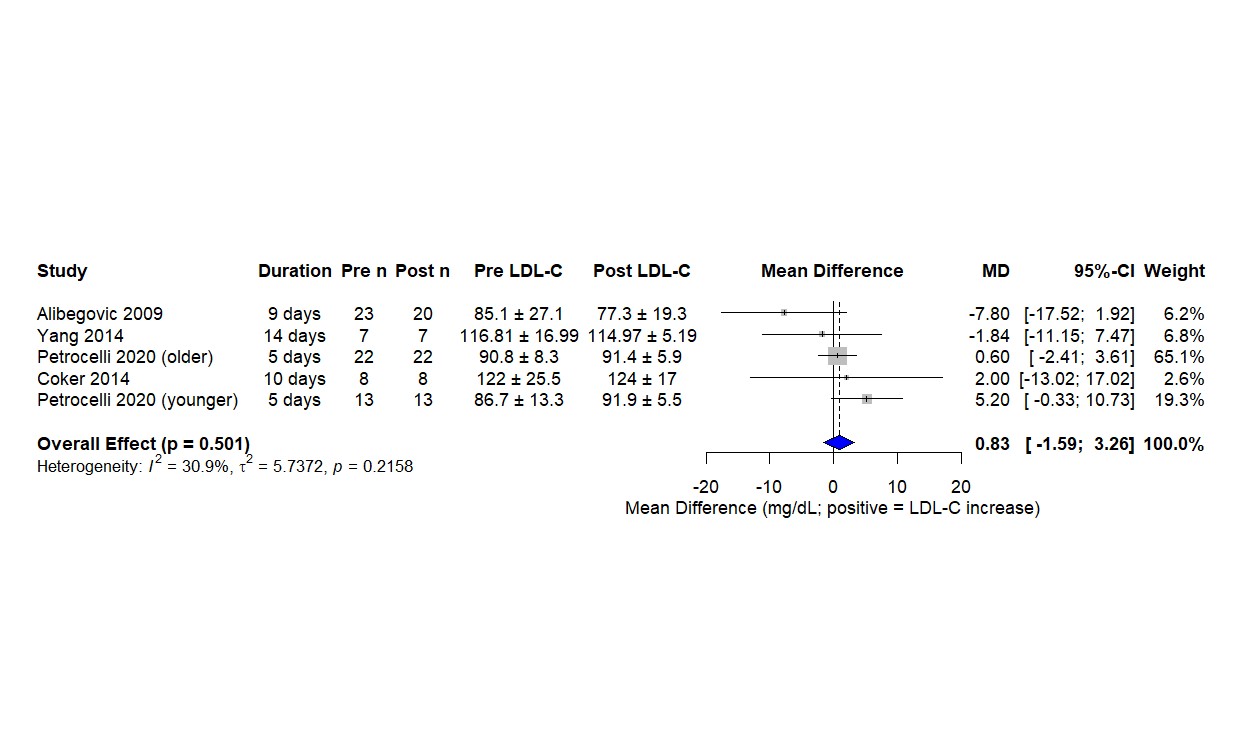


Head-down tilt (up to 60 days)


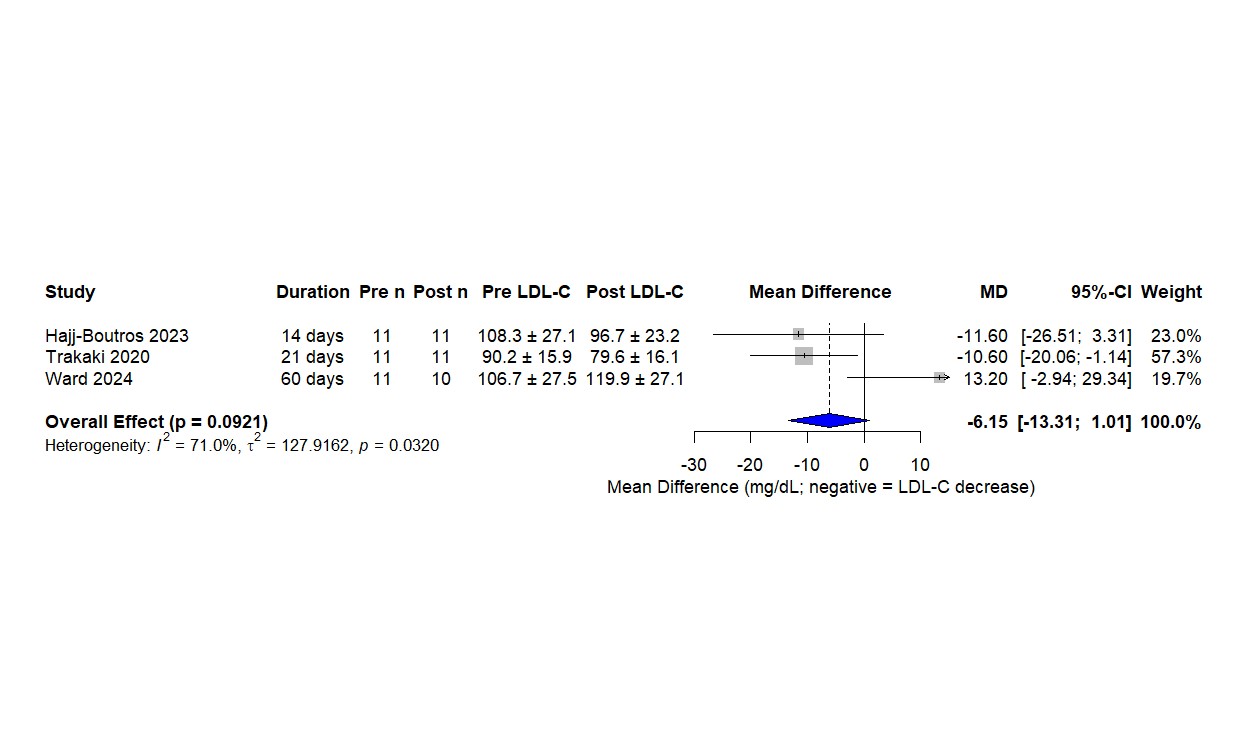


**Systolic blood pressure (mmHg)**

Bed rest (up to 10 days)

**
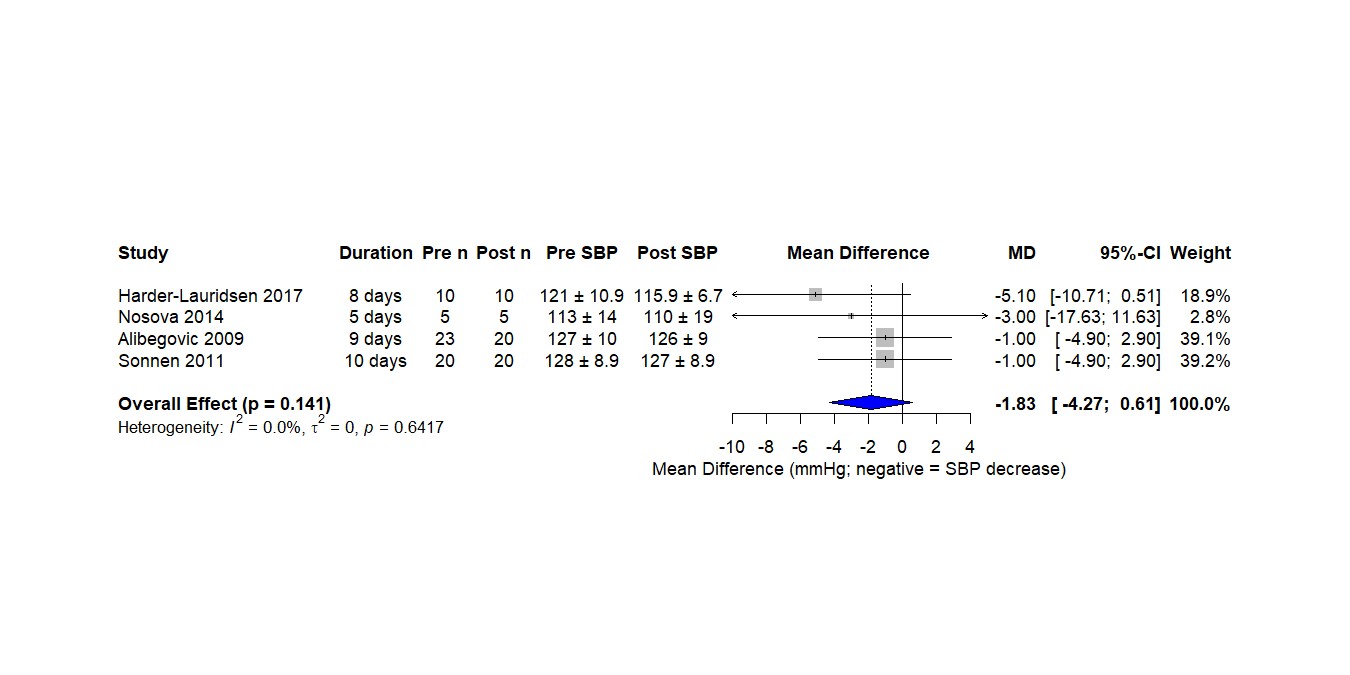
**

Head-down tilt (up to 14 days)

**
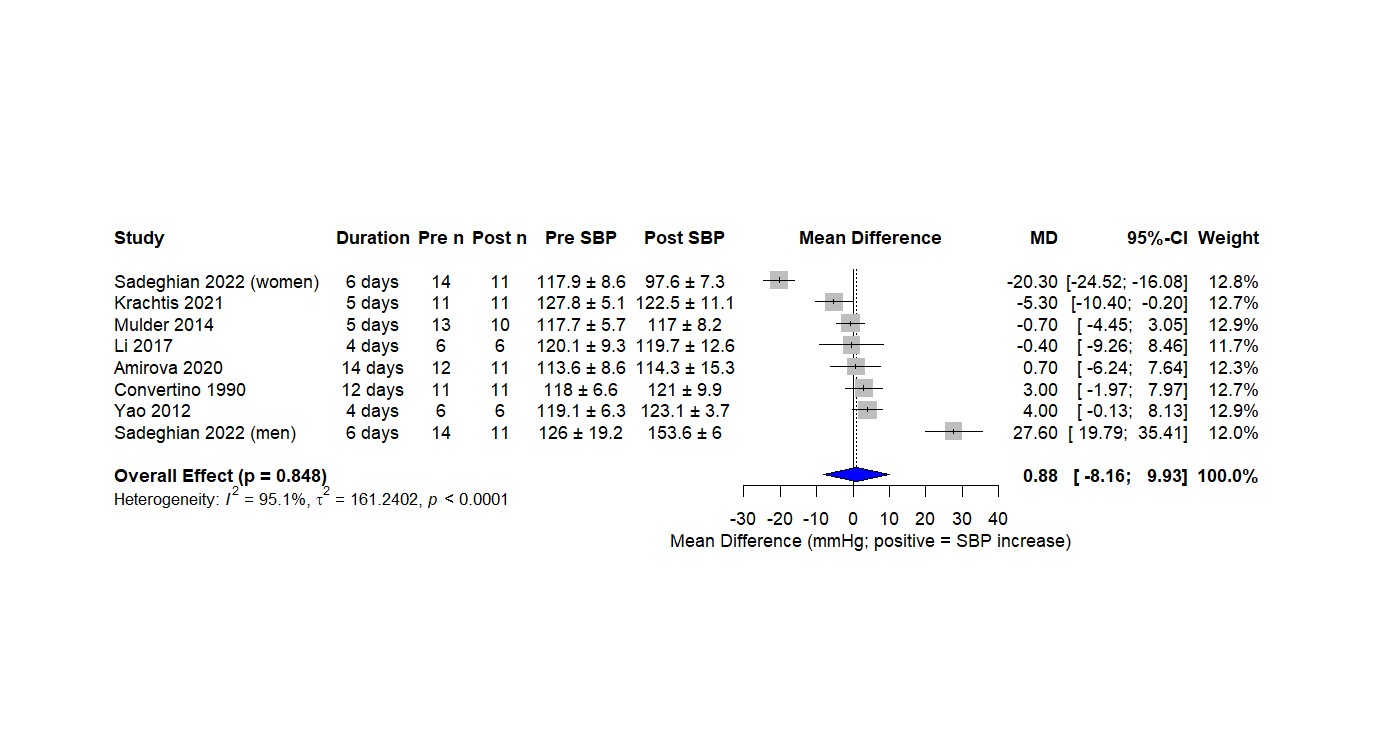
**

Head-down tilt (up to 30 days)

**
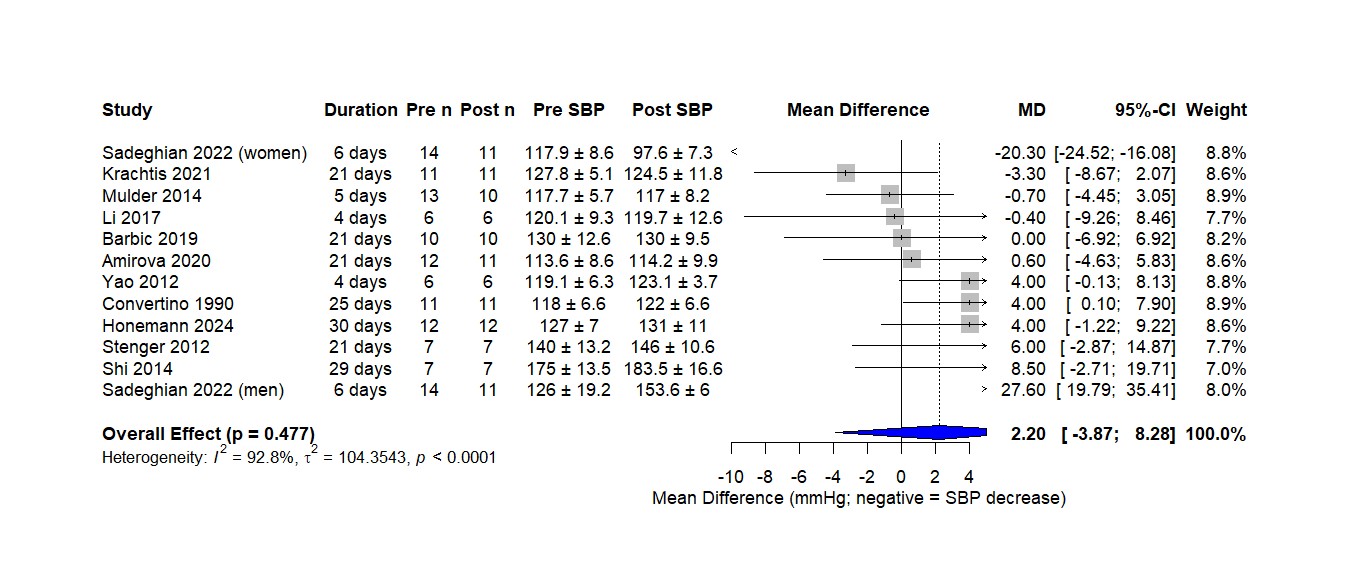
**

**Diastolic blood pressure (mmHg)**

Bed rest (up to 10 days)

**
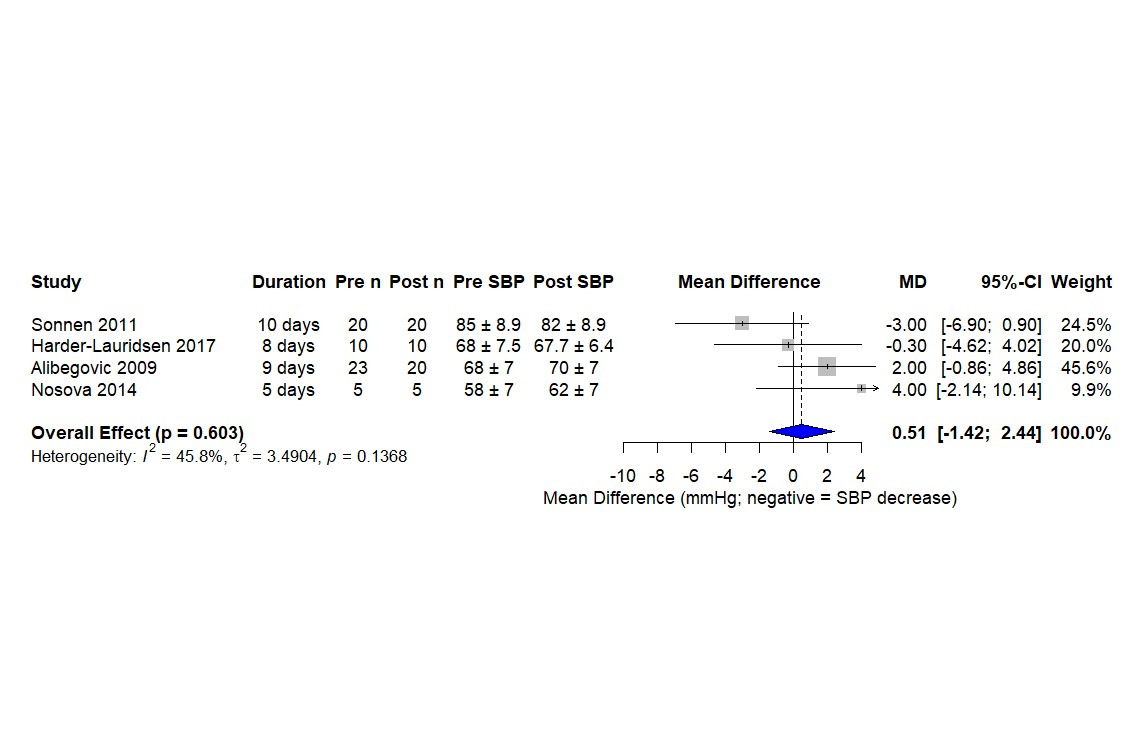
**

Head-down tilt (up to 14 days)

**
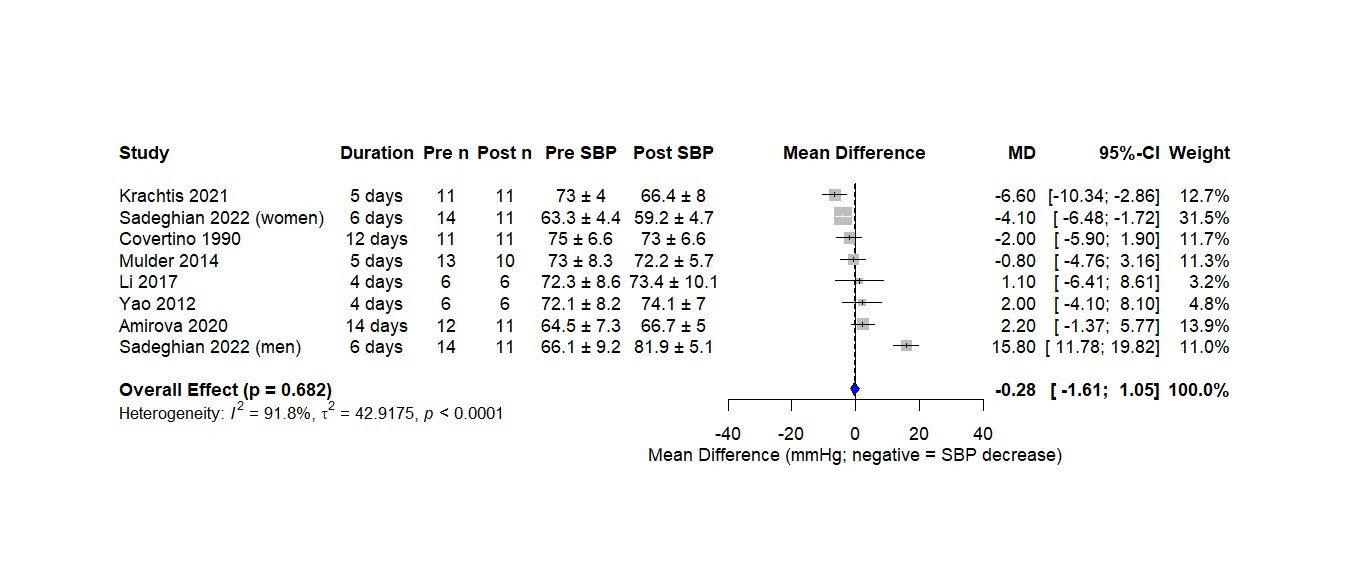
**

Head-down tilt (up to 30 days)

**
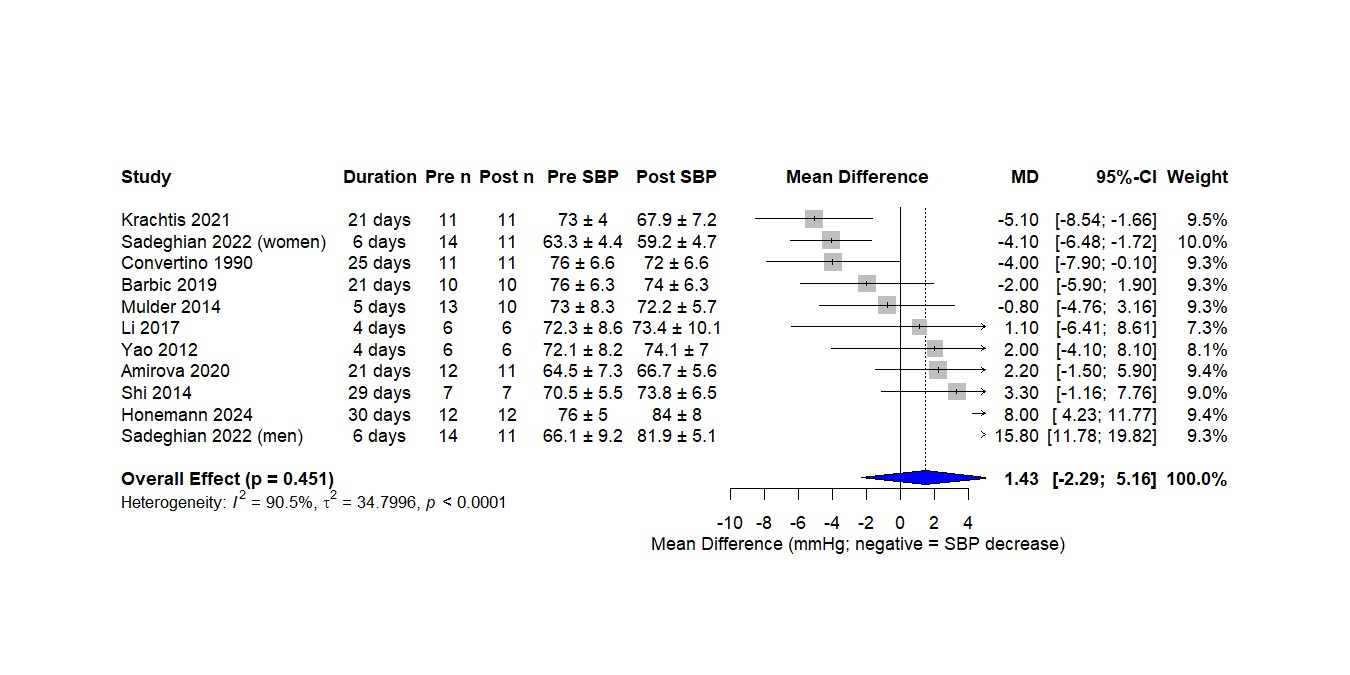
**

**C-reactive protein (mg/L)**

Bed rest (up to 14 days)

**
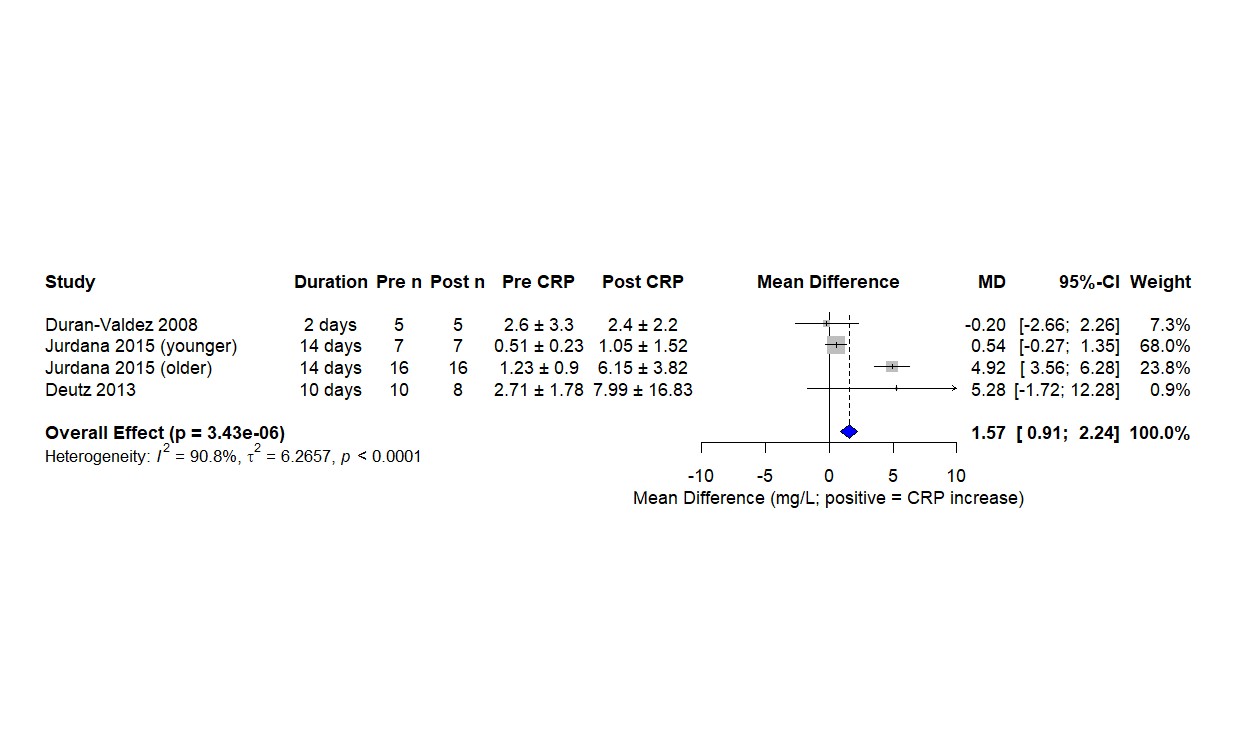
**

Head-down tilt (up to 55 days)

**
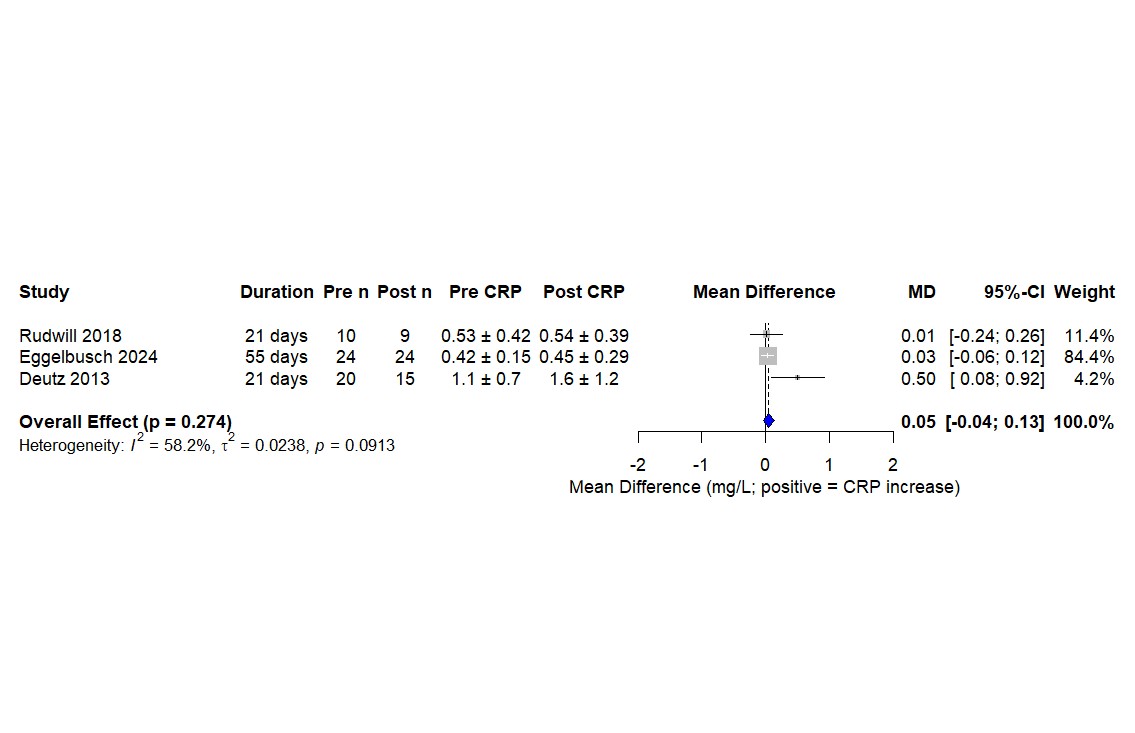
**

**Interleukin-6 (pg/mL)**

Bed rest (up to 14 days)

**
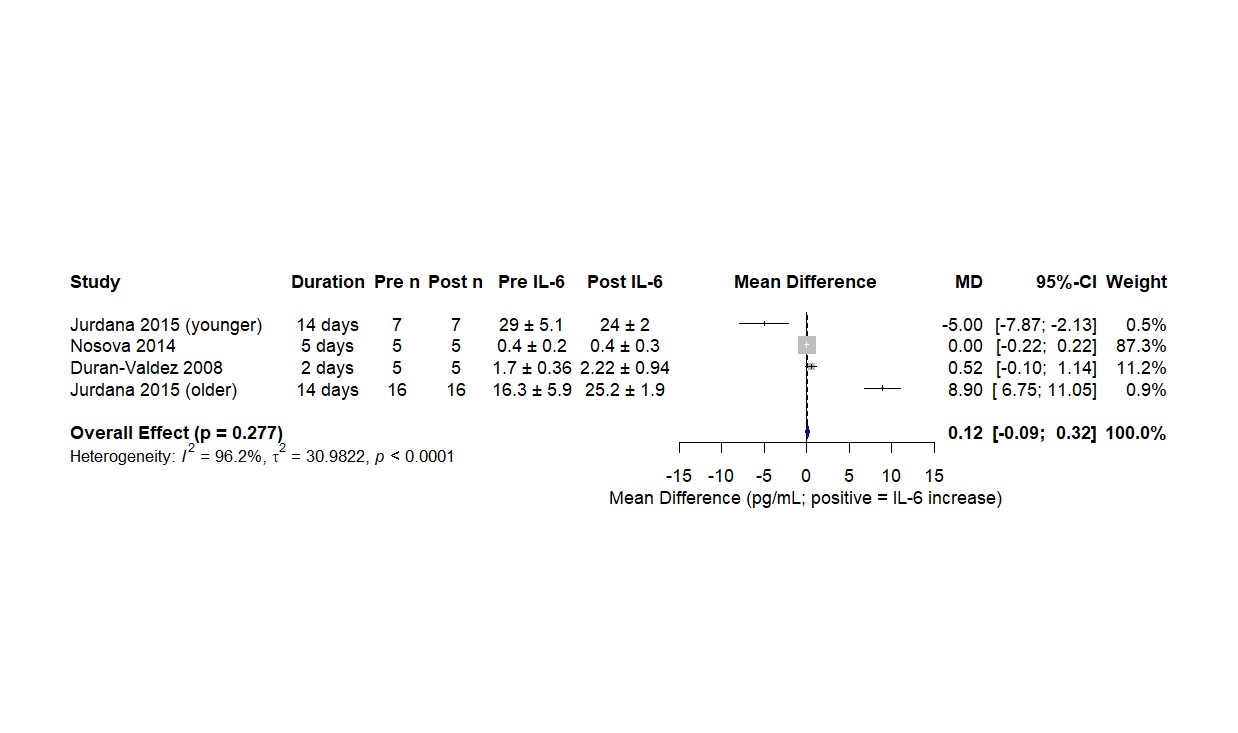
**

**Tumour necrosis factor-a (pg/mL)**

Bed rest (up to 14 days)

**
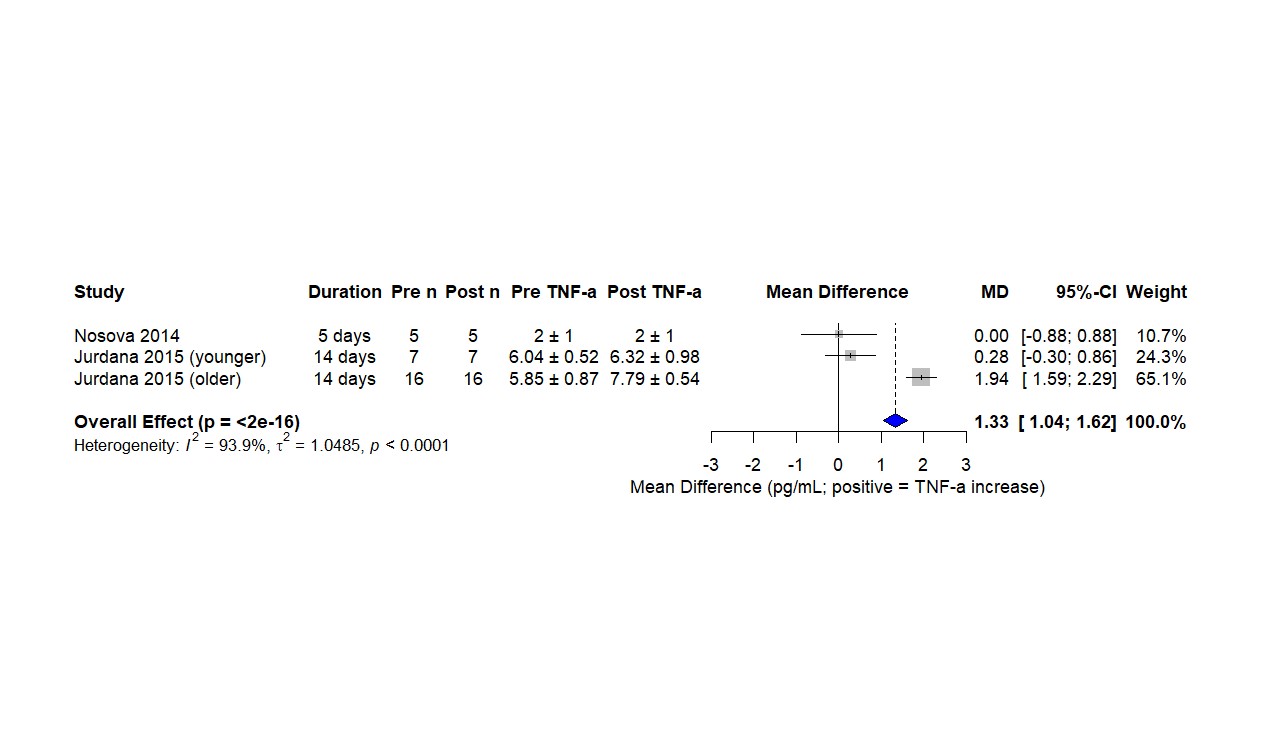
**

Head-down tilt (up to 14 days)

**
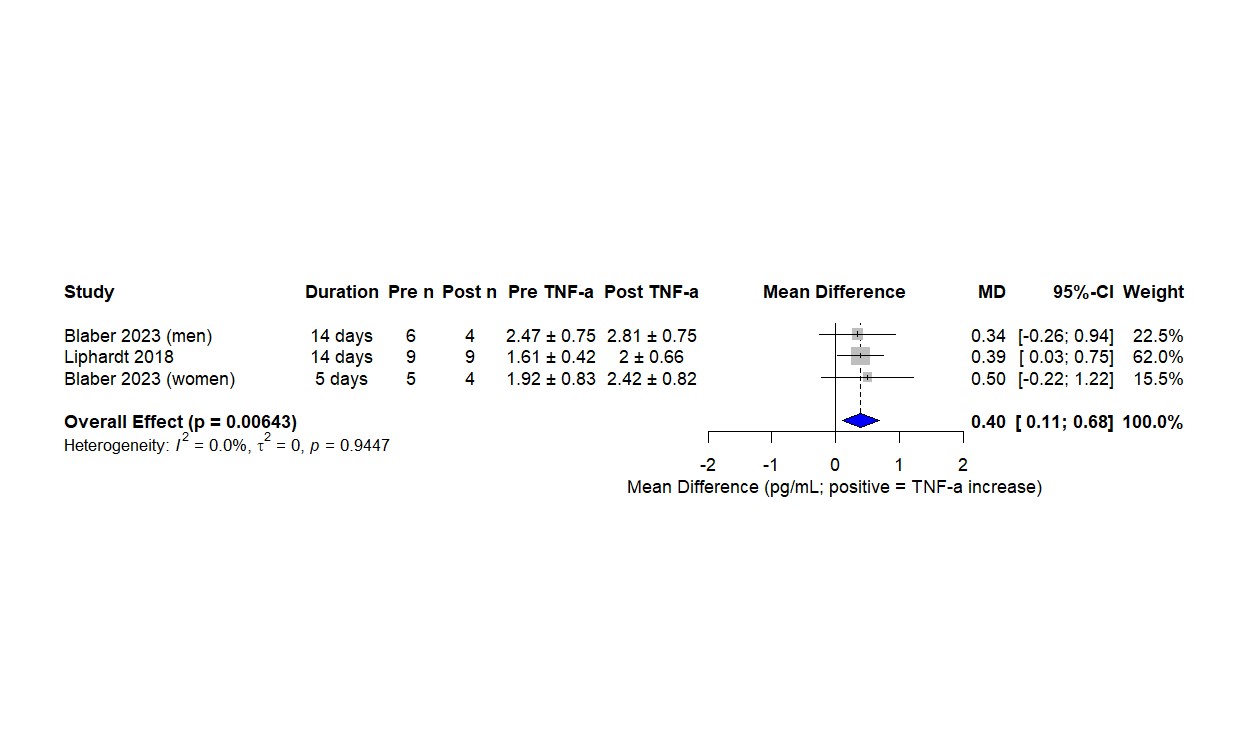
**
