# Supplementary material for: The effects of bed rest on cardiometabolic health: A systematic review and meta‐analysis
Source: Exp Physiol. 2026 Mar 19:10.1113/EP092944. Online ahead of print. doi: 10.1113/EP092944 (PMC13394784; doi:10.1113/EP092944)
Supplement: Supplementary file 2 — Supporting Information [file EPH-9999-0-s001.docx]

**Supplementary File 2.** Bubble plots.

**Body mass index/Bodyweight**

**
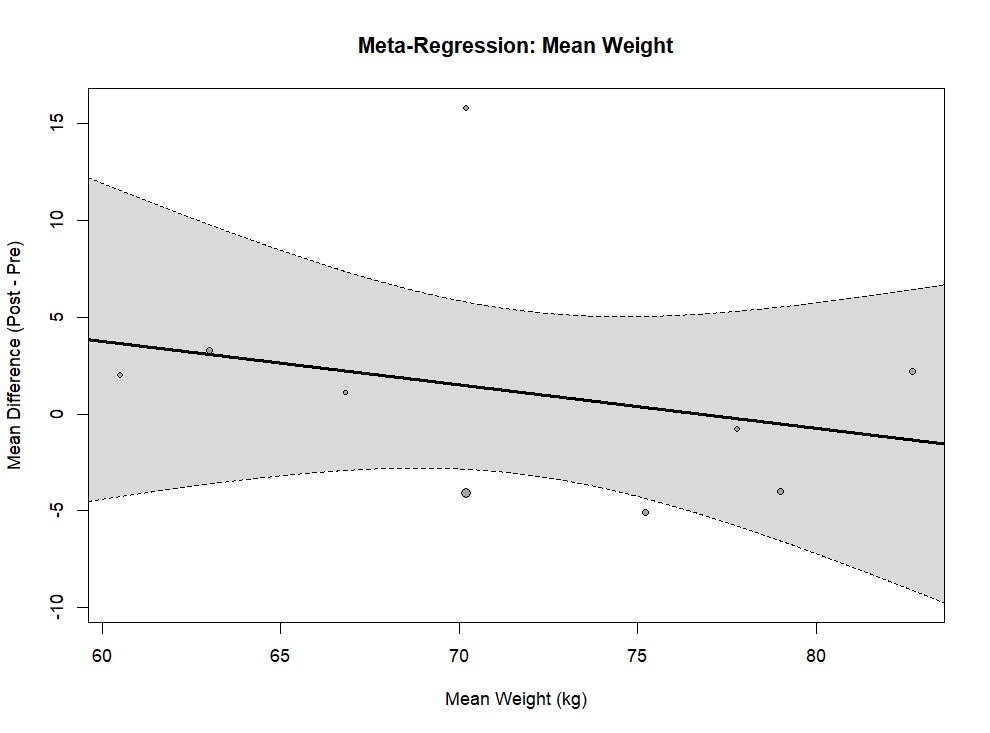
Diastolic blood pressure**

**Systolic blood pressure
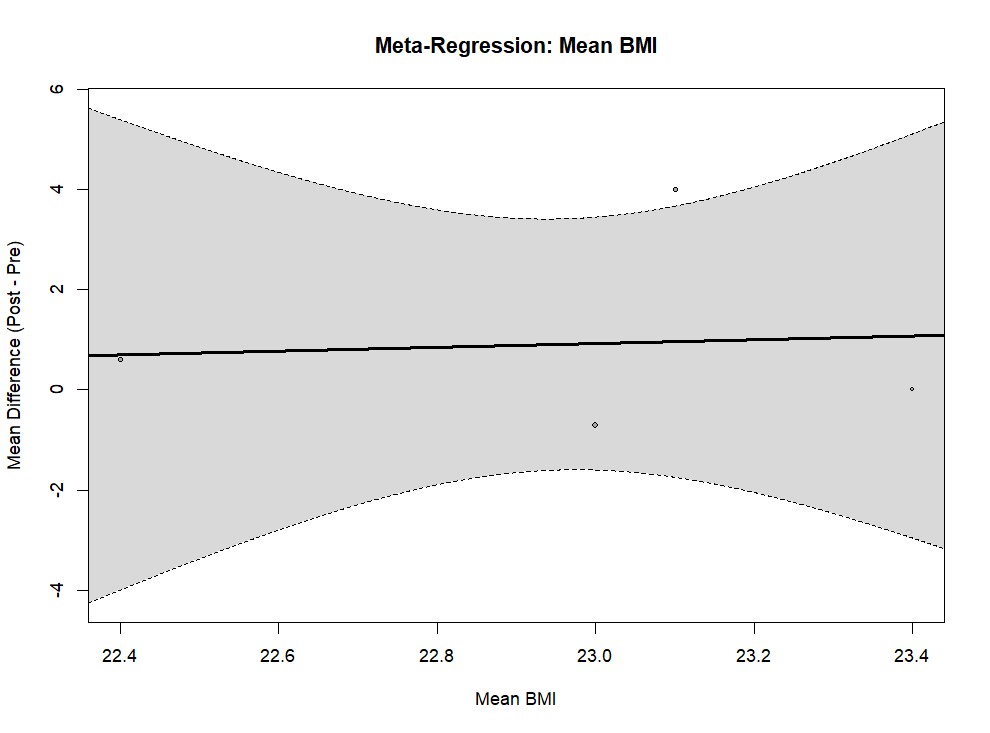
**

**
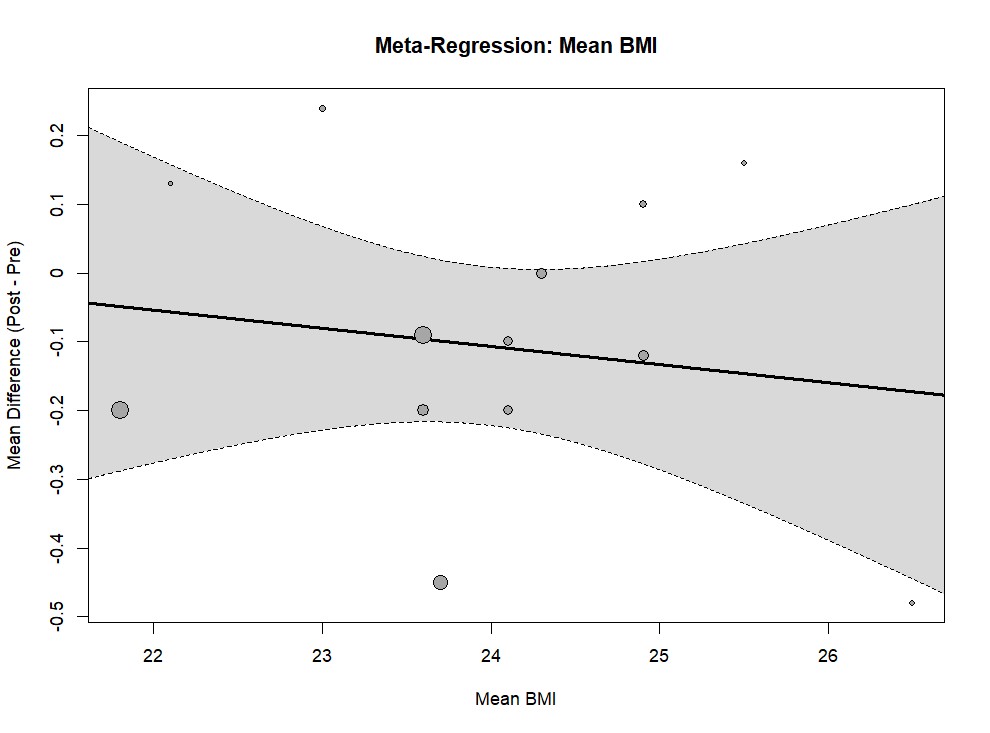
Glucose**

**Age**

**
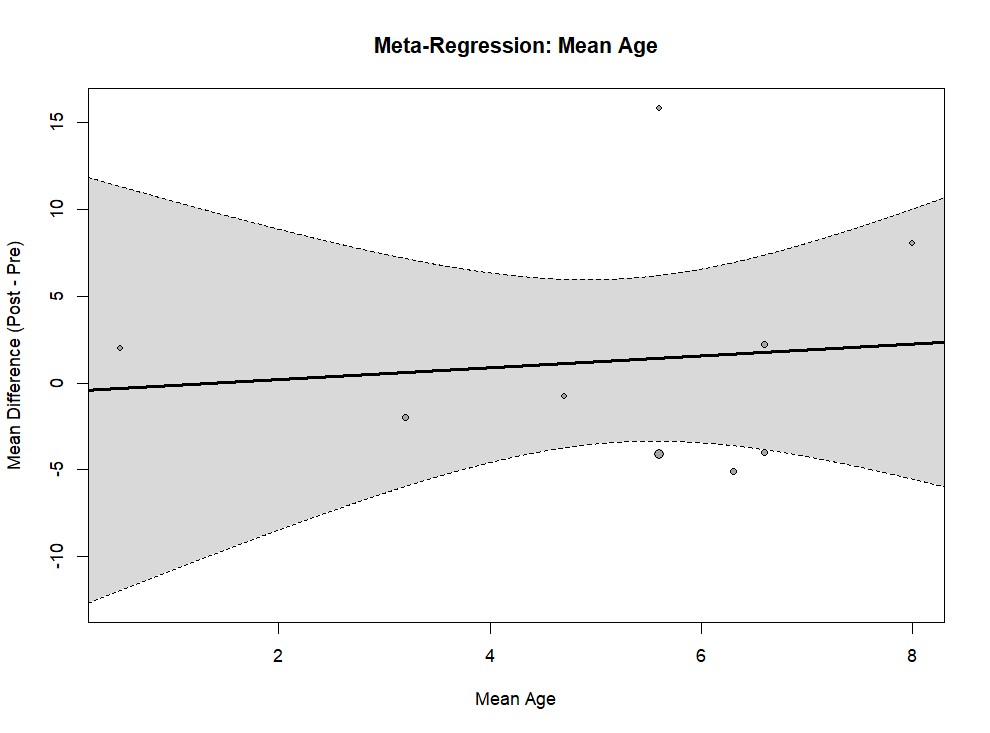
Diastolic blood pressure**

**
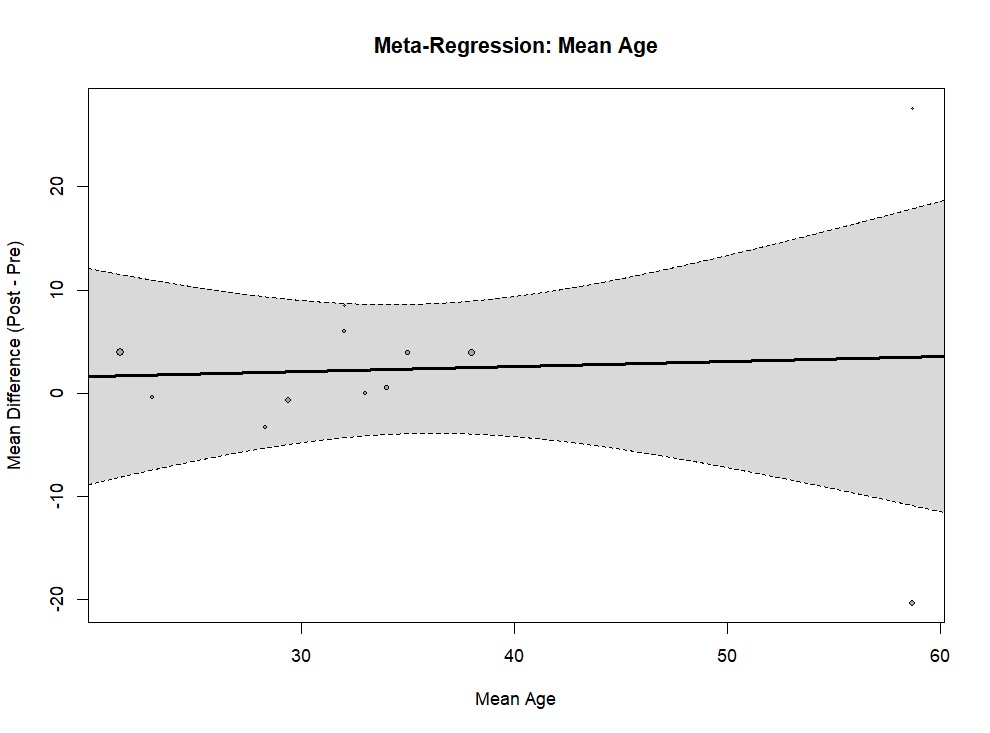
Systolic blood pressure**

**
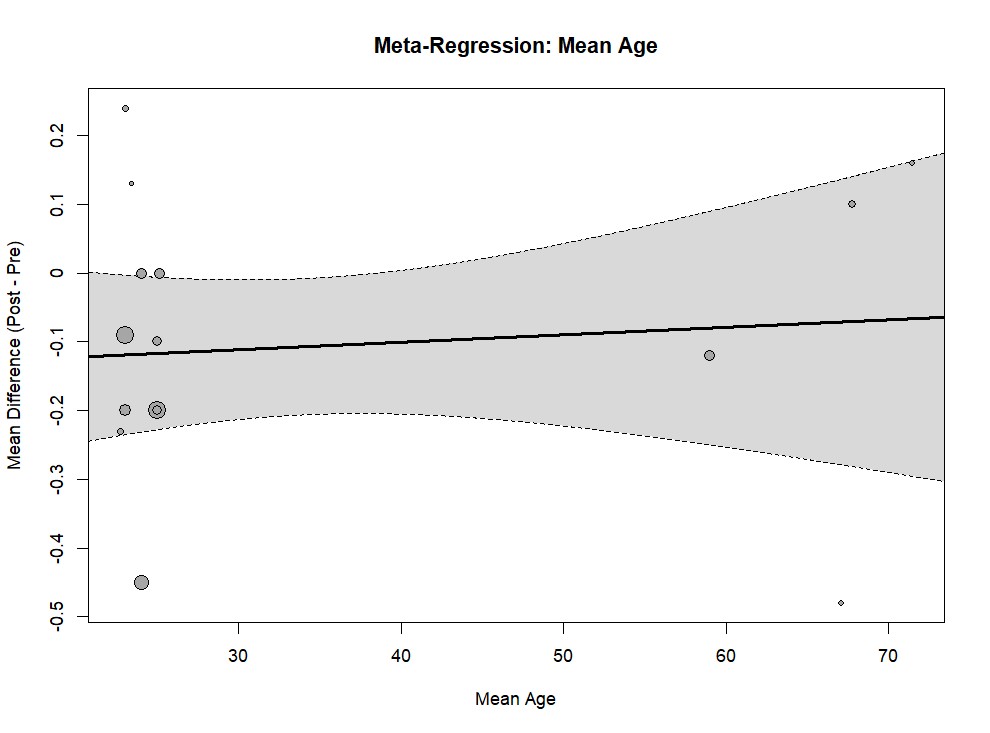
Glucose**

**Proportion of females**

**
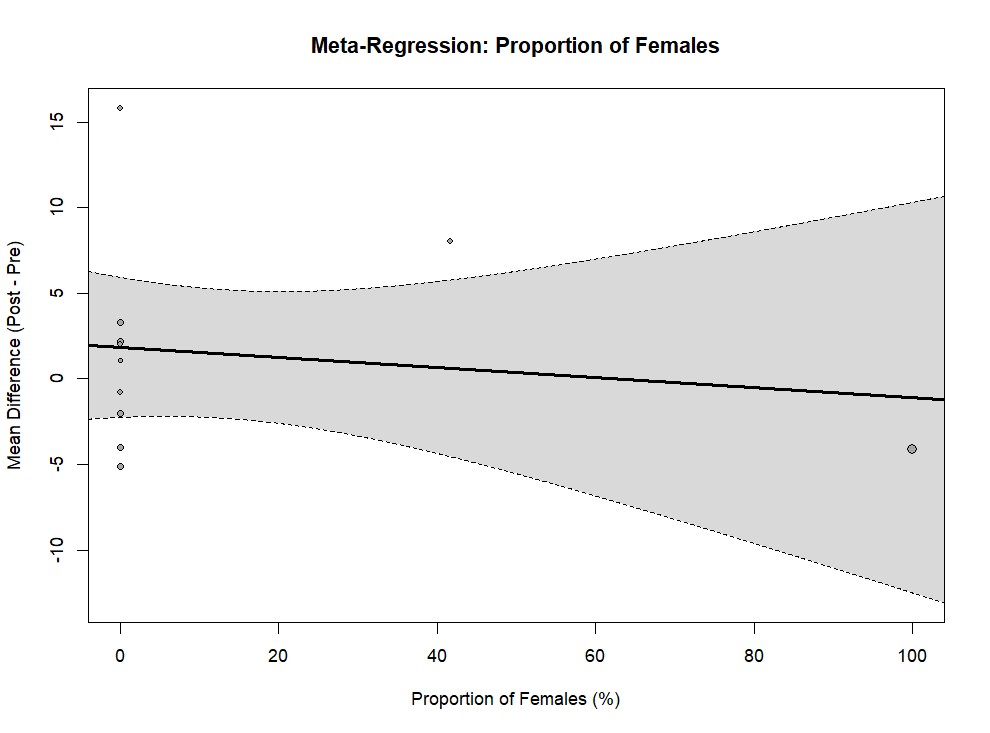
Diastolic blood pressure**

**
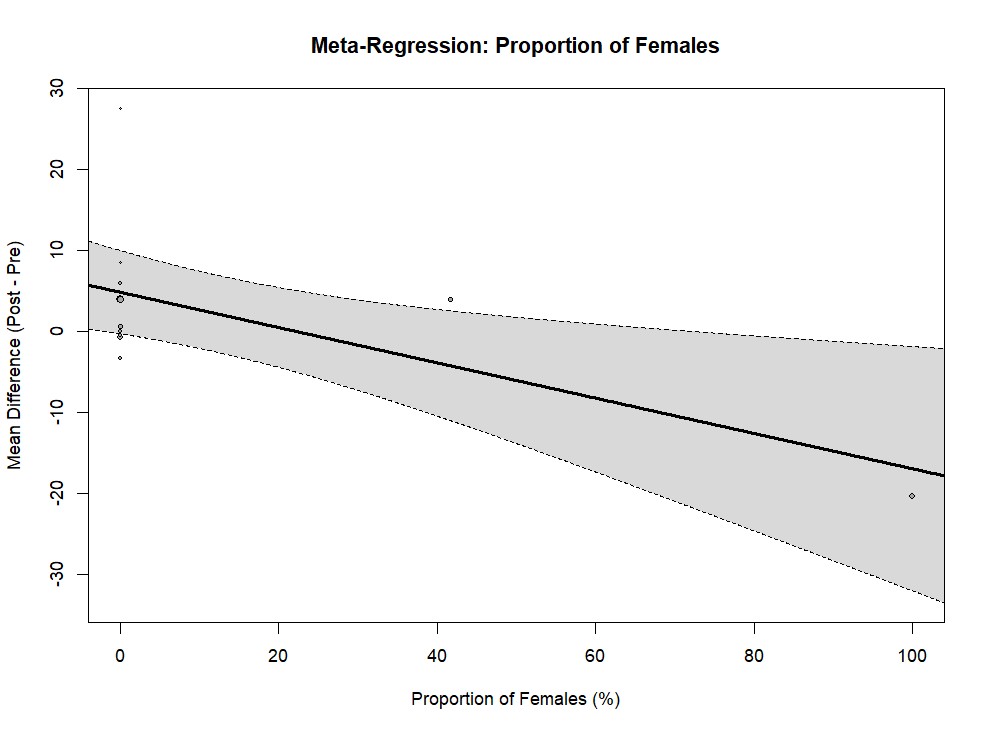
Systolic blood pressure**

**
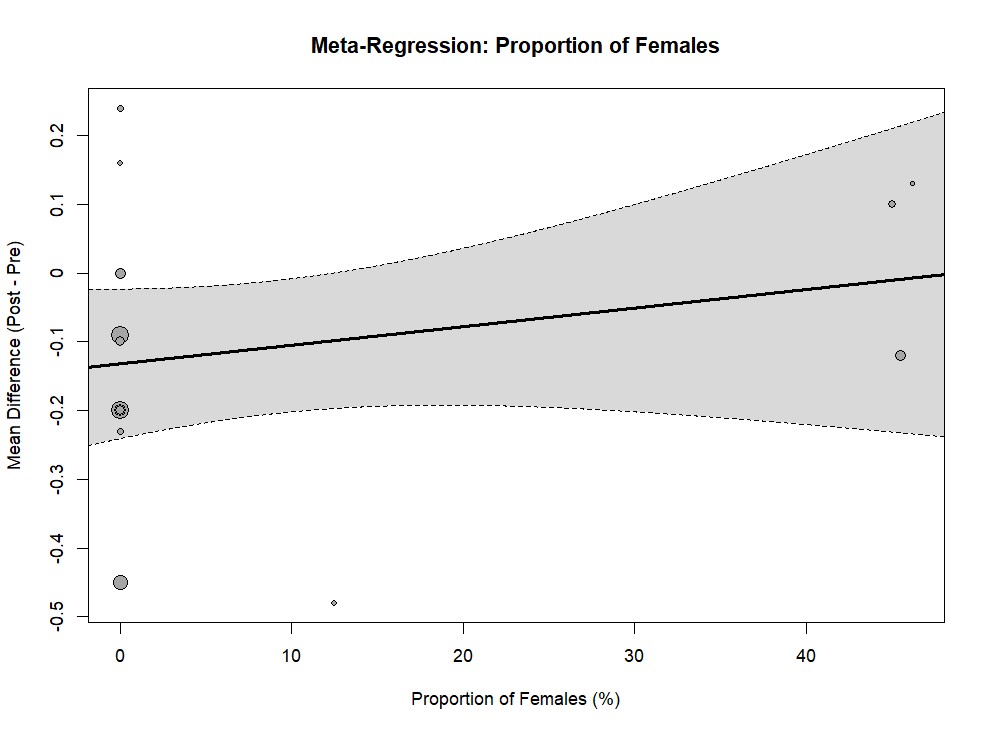
Glucose**
